# Microbial and Metabolomic Variations Correlated With Gastric Cancer Subtypes and Prognosis

**DOI:** 10.1002/mbo3.70139

**Published:** 2025-11-10

**Authors:** Yan Yang, Liping Wen, Wu Lin, Yiran Chen, Rui Yang, Chao He, Yingzi Zhang, Jing Zhang, Haohao Wang, Haiyong Wang, Lisong Teng

**Affiliations:** ^1^ Department of Surgical Oncology, the First Affiliated Hospital Zhejiang University School of Medicine Hangzhou 310003 China; ^2^ Department of Colorectal Surgery and Oncology (Key Laboratory of Cancer Prevention and Intervention China National Ministry of Education, Key Laboratory of Molecular Biology in Medical Sciences Zhejiang Province China; ^3^ The Second Affiliated Hospital, Zhejiang University School of Medicine Hangzhou Zhejiang China

**Keywords:** gastric cancer, Lauren classification, metabolome, microbiome, ZJU classification

## Abstract

Gastric cancer (GC) persists as the third most prevalent malignancy in China. GC exhibits distinct features when stratified by Lauren/ZJU subtypes. The interdependence of microbes, metabolites, and tumor evolution is recognized. Nevertheless, the specific microbial and metabolite disparities related to the Lauren and ZJU subtypes of GC have yet to be thoroughly investigated. In this study, we employed 16S sequencing of microbial communities and conducted untargeted metabolomic assessments on tumor tissues and their matched normal controls from 50 GC patients. We observed variations in microbial composition and metabolite landscapes across subtypes, irrespective of the Lauren or ZJU classification. We explored the associations and differences between the Lauren and the ZJU classification. It was found that both classifications share differential microbiota, including *Fusobacterium* and *Haemophilus*. Additionally, 38 of the top 50 differential metabolites are common to both classifications. However, distinct classifications also exhibit unique differential microbiota and metabolite characteristics. Among them, *Eubacterium_ventriosum_group* and N6‐Succinyl Adenosine are both characteristic differences of the ZJU classification. Multivariate survival analysis disclosed that *Eubacterium_ventriosum_group* positively correlates with poor prognosis, whereas N6‐Succinyl Adenosine negatively correlates with poor prognosis. Our research delineates the microbiota and metabolites specific to different subtypes of GC and investigates the interplay between these differential elements, as well as their prognostic significance. We have identified two distinct features that are both associated with the ZJU classification, suggesting that the ZJU classification is more closely related to prognosis.

## Graphical abstract

Multi‐omics analysis of 50 GC patients revealed conserved microbial‐metabolite signatures across Lauren/ZJU subtypes, with ZJU‐specific features—*Eubacterium_ventriosum_group* and N6‐Succinyl Adenosine—enabling superior prognostic stratification.

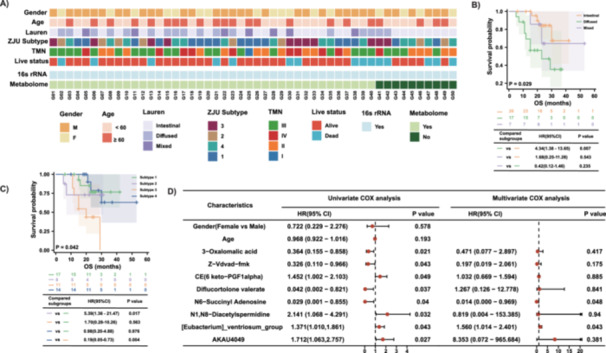

## 1 Introduction

Gastric cancer (GC) ranks fifth in common cancers and third in cancer deaths globally (Machlowska et al. 2020). Despite a decline in global incidence, GC remains prevalent in China. It shows high heterogeneity in molecular and phenotypic traits. Early‐stage GC is primarily treated with endoscopic resection. Advanced GC is mainly treated with a combination of radiation and chemotherapy, with a median survival of less than 1 year (Smyth et al. 2020).

GC has multiple classification methods. Based on histological differences, it is classified into intestinal and diffuse types according to the Lauren classification. A mixed type, showing features of both, has also been identified. Earlier studies indicate that diffuse‐type GC is mostly poorly differentiated and has a poorer prognosis (Zhang et al. 2021). Several molecular subtyping schemes have been proposed based on comprehensive molecular profiling data by The Cancer Genome Atlas (TCGA) and Asian Cancer Research Group (ACRG). TCGA categorized GC into Epstein–Barr virus (EBV) associated, microsatellite instable (MSI), genomically stable (GS), and chromosomal instability (CIN) subtypes, while the ACRG classified GC into microsatellite stable (MSS)/epithelial–mesenchymal transition (EMT), MSI, MSS/p53 +, and MSS/p53− subtypes (Puliga et al. 2021). Based on whole‐exome profiling, we have also identified four GC subtypes (known as the ZJU classification) that not just represent distinct mutational signatures but also closely correlate to phenotypic manifestation and prognosis (Wang 2021a). The ZJU Subtype 1 features recurrent *TP53* mutation, *ERBB2* amplification, and high tumor mutation burden (TMB)/tumor neoantigen burden (TNB) and exhibits intratumoral heterogeneity and liver metastasis tendency. Subtype 2 has frequent *TP53*/*SYNE1* co‐mutations and high TMB/TNB and usually are elderly‐onset GC with poor prognosis. Subtypes 3 and 4 exhibit genomic/chromosomal stability, but each bears a distinct set of mutational features. They both have a peritoneal metastasis tendency, but Subtype 3 correlates with poor prognosis while Subtype 4 correlates with favorable prognosis. Intriguingly, the ZJU classification could also link to the Lauren classification, suggesting an inherent connection between genomic variations and histological appearances. For example, Subtype 1 is intestinal‐type dominant, whereas subtypes 3 and 4 are mostly diffuse/mixed‐type (Wang 2021a).

The relationship between microbiota and tumor development has been extensively studied (Dai et al. 2021). Significant differences in tissue microbiota have been observed among individuals with peritumor, tumor, and normal tissues (Liu et al. 2019). Previous studies have provided preliminary insights into the microbial differences based on the Lauren classification of gastric cancer, but a detailed exploration is lacking. Similarly, numerous studies have investigated the relationship between metabolites and tumor progression (Xu et al. 2023; Sun et al. 2023). However, the microbial and metabolic differences specific to different subtypes of gastric cancer have not been fully elucidated. Our study aims to elucidate differential microbiota and metabolites associated with the Lauren and ZJU classifications and to assess their correlation with prognosis. The objective is to deepen understanding of subtype variations in gastric cancer, with the aim of contributing to future refinements in gastric cancer classification.

## 2 Methods

### 2.1 Samples

50 Gastric cancer (GC) patients with no history of preoperative chemotherapy were enrolled from January 2015 to August 2019 at the First Affiliated Hospital, School of Medicine, Zhejiang University. All GC patients were diagnosed by postoperative pathological examinations. The Lauren classification, ZJU classification, and other clinical‐pathological features are listed in Table 1.

**Table 1:** The clinicopathological characteristics of patient cohort (*n* = 50).

Patient ID	Gender	Age	TNM	Lauren classification	ZJU‐GC subtype	Live status	Overall survival (months)	16 s rRNA gene sequencing	metabolome analysis
G01	M	69	IIIC	Intestinal	3	Alive	24	Yes	Yes
G02	F	62	IIIA	Diffused	2	Dead	7	Yes	Yes
G03	M	49	IIIB	Diffused	4	Alive	37	Yes	Yes
G04	M	64	IIIB	Intestinal	1	Alive	24	Yes	Yes
G05	M	63	IV	Mixed	1	Alive	24	Yes	Yes
G06	M	38	IIIB	Diffused	3	Dead	29	Yes	Yes
G07	M	73	IIB	Intestinal	2	Alive	29	Yes	Yes
G08	F	58	IIB	Diffused	4	Alive	10	Yes	Yes
G09	M	76	IIIB	Intestinal	2	Alive	6	Yes	Yes
G10	M	62	IIB	Diffused	4	Alive	32	Yes	Yes
G11	F	45	IIIC	Mixed	1	Alive	25	Yes	Yes
G12	M	80	IIIB	Intestinal	2	Alive	31	Yes	Yes
G13	M	58	IIIB	Diffused	3	Dead	12	Yes	Yes
G14	M	66	IV	Intestinal	1	Alive	6	Yes	Yes
G15	F	51	IIIB	Diffused	3	Alive	15	Yes	Yes
G16	F	41	IIIB	Diffused	4	Alive	28	Yes	Yes
G17	M	42	IV	Diffused	4	Alive	20	Yes	Yes
G18	M	62	IV	Intestinal	1	Alive	6	Yes	Yes
G19	F	43	IIA	Diffused	1	Alive	36	Yes	Yes
G20	F	65	IIIA	Intestinal	1	Alive	24	Yes	Yes
G21	M	44	IIIA	Diffused	1	Dead	15	Yes	Yes
G22	M	55	IIA	Mixed	4	Alive	53	Yes	Yes
G23	M	53	IV	Diffused	2	Dead	5	Yes	Yes
G24	M	69	IIIB	Intestinal	1	Alive	23	Yes	Yes
G25	M	59	IB	Mixed	1	Alive	22	Yes	Yes
G26	F	59	IIB	Intestinal	1	Alive	61	Yes	Yes
G27	M	68	IIA	Intestinal	1	Alive	42	Yes	Yes
G28	F	63	IIIA	Mixed	4	Alive	21	Yes	Yes
G29	F	61	IIB	Intestinal	2	Alive	22	Yes	Yes
G30	F	49	IV	Mixed	3	Dead	15	Yes	Yes
G31	M	60	IV	Intestinal	3	Dead	20	Yes	Yes
G32	M	62	IIA	Intestinal	3	Alive	20	Yes	Yes
G33	F	62	IIA	Intestinal	3	Alive	25	Yes	Yes
G34	M	46	IV	Intestinal	4	Alive	19	Yes	Yes
G35	F	51	IIIC	Diffused	4	Alive	18	Yes	Yes
G36	M	71	IV	Intestinal	1	Alive	12	Yes	Yes
G37	M	58	IIA	Intestinal	1	Alive	15	Yes	Yes
G38	M	70	IIIA	Mixed	1	Dead	23	Yes	Yes
G39	M	61	IIIC	Diffused	4	Dead	23	Yes	Yes
G40	M	48	IIIC	Diffused	3	Dead	15	Yes	Yes
G41	F	50	IV	Diffused	3	Dead	11	Yes	No
G42	M	69	IIIB	Diffused	3	Dead	11	Yes	No
G43	M	64	IIIC	Intestinal	1	Alive	26	Yes	No
G44	F	60	IIIB	Intestinal	4	Alive	28	Yes	No
G45	M	66	IIA	Intestinal	4	Alive	30	Yes	No
G46	F	57	IIB	Intestinal	2	Alive	29	Yes	No
G47	M	65	IIB	Intestinal	4	Dead	30	Yes	No
G48	M	42	IIIC	Intestinal	1	Dead	16	Yes	No
G49	M	55	IIB	Intestinal	2	Alive	19	Yes	No
G50	F	64	IIB	Intestinal	4	Dead	21	Yes	No

F, female; M, male.

### 2.2 Dna Extraction, 16S Detection, and Data Analysis

Gastric cancer tissues and normal control tissues from 50 gastric cancer patients were used for 16S detection. DNA from all tissues was extracted using Qiagen's genomic extraction kit (Yang 2022a). DNA concentration, purity, and integrity were assessed using a NanoDrop spectrophotometer (A260/280: 1.7–1.9; A260/230: > 2.0) and 0.8% agarose gel electrophoresis, confirming sharp high‐molecular‐weight bands (> 10 kb) without degradation smears or RNA contamination. The V3‐V4 variable regions of 16S rRNA genes were amplified with universal primers 343 F and 798 R. Finally, the library was sequenced on an Ion S5TM XL platform, and 400–600 bp single‐end reads were generated.

Raw sequencing data were in the FASTQ format. QIIME2 software was utilized to investigate species diversity and evaluate differences in microbial community composition (Bolyen et al. 2019). At last, the software outputs the representative reads and the ASV abundance table. Bioinformatics analysis was performed with the OECloud tools at https://cloud.oebiotech.cn.

### 2.3 Metabolome Detection and Data Analysis

40 pairs of gastric cancer tissues and normal control tissues were used to extract metabolites. Sample preparation was performed using ice‐cold solvents under temperature‐controlled conditions. Briefly, 100 μL aliquots were homogenized in 600 μL of pre‐chilled methanol‐water (4:1 v/v, containing internal standards) by pipette dispersion, followed by ice‐bath sonication (1620 W, 10 min; 6 s on/4 s off cycles). After a 20‐min ice‐water bath extraction, samples were incubated at −40°C overnight. Subsequent centrifugation (12,000 rpm, 4°C, 10 min) yielded supernatants that were dried under nitrogen. Pellets were reconstituted in 300 μL methanol‐water (1:4 v/v), vortexed (1 min), sonicated (10 min), and re‐incubated at −40°C overnight. Final centrifugation (12,000 rpm, 4°C, 20 min) provided 150 μL supernatants for analysis in LC‐MS vials with insert feet. Quality control (QC) samples were prepared by pooling equal volumes of all extracts.

Chromatographic separation was achieved using a Waters ACQUITY UPLC I‐Class Plus system equipped with an HSS T3 column (100 × 2.1 mm, 1.8 μm) maintained at 45°C. Mobile phases consisted of (A) 0.1% formic acid in water and (B) acetonitrile at a 0.35 mL/min flow rate, with 5 μL injections. Metabolite detection employed a Thermo Q Exactive mass spectrometer with HESI ion source operating in both positive/negative modes. Data acquisition utilized data‐dependent analysis (DDA) with Full MS/dd‐MS² (TOP 10) scanning (Zhang et al. 2023).

LC‐MS raw data were processed in Progenesis QI v2.3 (baseline correction, peak alignment with 5 ppm/10 ppm tolerances, normalization). Metabolites were identified via m/z, MS/MS, and isotopes using HMDB/LipidMaps/METLIN/in‐house databases. Features with > 50% missing values were excluded; zeros were imputed with half‐minima; compounds scoring < 36/60 were discarded. PCA/OPLS‐DA/PLS‐DA in R (validated by sevenfold cross‐validation/RPT‐200) identified differential metabolites (VIP > 1, *p* < 0.05, *t*‐test).

### 2.4 Statistical Analysis

T‐test and one‐way ANOVA were performed to compare the differences between groups. Kaplan–Meier curves were examined using a log‐rank test. The hazard ratio (HR) with corresponding 95% confidence interval (CI) was determined using Cox regression analysis. Two‐sided P‐values were used throughout the analyses. *p*‐value < 0.05 was considered statistically significant. All statistical analyses were performed using SPSS version 26.0 (IBM, USA) and R software (version 3.6.3).

### 2.5 Combined Microbiome‐Metabolome Analysis

Microbiome‐metabolome correlation analysis was conducted using Spearman correlation analysis with paired 16S data and untargeted metabolomics data.

## 3 Results

### 3.1 Microbial Diversity in Lauren‐Typed Gastric Cancer

**Figure 1:**
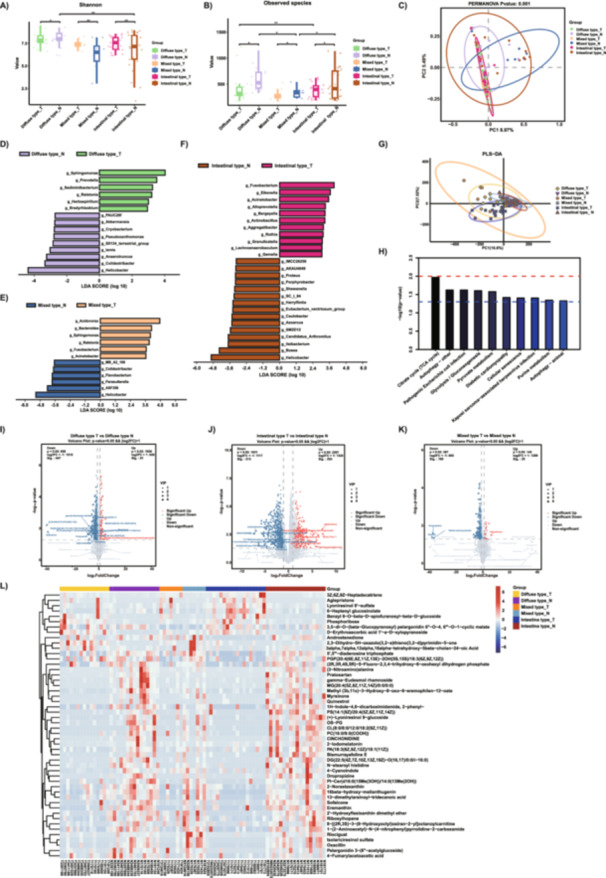
The differences in microbiota and metabolites among the three subtypes of Lauren classification. (A, B) The Shannon and Observed species were used to evaluate the microbial diversity of the three subtypes of Lauren classification. (C) PCoA of weighted UniFrac distance demonstrated that the three subtypes of tumor tissues and matched nontumor tissues showed distinct clusters. (D–F) Differential taxa at genus level identified by LefSe analysis (LDA ＞ 3.0, *Q* ＜ 0.05). (G) PLS‐DA showed that three subtypes of tumor tissues and matched nontumor tissues were separated into six clusters. H) The KEGG metabolic pathway enrichment chart shows the main enriched pathways (*p* < 0.05) for six groups of differentially abundant metabolites. (I–K) Volcano map of different metabolites between three subtypes of tumor tissues and matched nontumor tissues (VIP > 1 and *p* value < 0.05). L) Heatmap represents differential metabolites between six groups. Diffuse type_T: Diffuse type GC tumor tissues; Diffuse type_N: Diffuse type GC normal tissues; Intestinal type_T: Intestinal type GC tumor tissues; Intestinal type_N: Intestinal type GC normal tissues; Mixed type_T: Mixed type GC tumor tissues; Mixed type_N: Mixed type GC normal tissues; The horizontal axis represents sample names, and the vertical axis represents differentially abundant metabolites. The color gradient from blue to red indicates the expression abundance of metabolites, with red indicating higher expression levels of differentially abundant metabolites. *p ≤ 0.05; **p ≤ 0.01; ***p ≤ 0.001.

Alpha diversity reveals significant variations between tumor and normal tissues across the three Lauren types. The Shannon index shows substantial differences between normal tissues of diffuse‐type and intestinal‐type gastric cancer patients (Figure 1A). The observed species index also indicates marked differences among normal tissues of the three gastric cancer types. Additionally, the observed species index highlights considerable differences between tumor tissues of diffuse‐type and intestinal‐type gastric cancer patients (Figure 1B). Weighted UniFrac PCoA further confirms these differences, with significant variations in confidence ellipses among the six groups (*p* = 0.007, Figure 1C).

We employed the LefSe analysis to identify differential microbiota between tumor tissues and normal control tissues across three gastric cancer types. At the genus level, the differential microbiota in diffuse‐type gastric cancer primarily included *Sphingomonas*, *Prevotella*, *Sediminibacterium*, *Ralstonia*, *Herbaspirillum*, *Bradyrhizobium*, PAUC26f, *Lampropedia*, *Akkermansia*, *Cryobacterium*, *Pseudoxanthomonas*, *S0134_terrestrial_group*, *Anaerotruncus*, *Colidextribacter*, and *Helicobacter* (Figure 1D). Conversely, the differential microbiota in intestinal‐type gastric cancer encompassed *Fusobacterium*, *Eikenella*, *Acinetobacter*, *Alloprevotella*, *Bergeyella*, *Actinobacillus*, *Aggregatibacter*, *Rohillaceae*, *Granulicatella*, *Lachnoanaerobaculum*, *Gemella*, IMCC26256, AKAU4049, *Proteus*, *Porphyrobacter*, *Shewanella*, SC_I_84, *Azoarcus*, *Candidatus_Arthromitus*, *Ileibacterium*, SM2D12, *Harryflintia*, *Eubacterium_ventriosum_group*, *Caulobacter*, *Bosea*, and *Helicobacter* (Figure 1F). The differential microbiota in mixed‐type gastric cancer comprised *Acidovorax*, *Bacteroides*, *Sphingomonas*, *Ralstonia*, *Fusobacterium*, *Acinetobacter*, MB_A2_108, *Colidextribacter*, *Flavobacterium*, *Parasutterella*, ASF356, and *Helicobacter* (Figure 1E).

We observed a significant number of similar differential microbes when comparing tumor and control tissues across the three subtypes. Consequently, we further compared the microbial differences among the three types. Alpha diversity analysis revealed no significant differences in the microbial Shannon index between tumor tissues of the three subtypes, but significant differences were observed in Observed species (Supporting Information: Figure S1A). Beta diversity analysis showed no significant differences in PCoA confidence intervals among the three groups (Figure S1B). LefSe analysis identified microbial biomarkers at the genus level, primarily including *Fusobacterium*, *Alloprevotella*, *Haemophilus*, *Bacteroides*, *Ralstonia*, *Sphingomonas*, and ELLIN6055 in tumor tissues of the three subtypes (Supporting Information: Figure S1C,D).

### 3.2 Distinct Metabolites Associated With the Lauren Classification of Gastric Cancer

Due to the differences in microbial composition between gastric cancer tumor tissues classified by the Lauren classification and normal control tissues, we hypothesize that the microbial–host interactions within the tissue microenvironment may contribute to differential tissue metabolism. Untargeted metabolomic sequencing results indicate significant metabolic differences between diffuse‐type gastric cancer tissues and normal control tissues.

PLS‐DA analysis revealed significant differences in the metabolism of gastric cancer tumor tissues of the Lauren classification compared to normal control tissues (Q2 = 0.121, R2 = 0.059) (Figure 1G). KEGG pathway analysis indicated that the differential metabolites of the three types of gastric cancer were mainly associated with pathways such as Citrate cycle (TCA cycle), Autophagy‐other, Pathogenic *Escherichia coli* infection, Glycolysis/Gluconeogenesis, Pyruvate metabolism, Diabetic cardiomyopathy, Cellular senescence, Kaposi sarcoma‐associated herpesvirus infection, Purine metabolism, and Autophagy‐animal (Figure 1H). There are 528 differential metabolites (VIP ≥ 1, log2FC ≥ 1 or ≤−1, *p* ≤ 0.05) identified between diffuse‐type GC tissues and normal control tissues. Similarly, there are 720 distinct metabolites between intestinal‐type GC tissues and normal control tissues. In mixed‐type gastric cancer, there are 418 distinct metabolites between cancer and normal control tissues. The Volcano plots displayed the differential metabolites between diffuse‐type gastric cancer tumor tissues and control tissues, intestinal‐type gastric cancer tumor tissues and normal control tissues, as well as mixed‐type gastric cancer and control tissues (*p* ≤ 0.05, VIP ≥ 1) (Figure 1I–K). It can be observed from the Volcano plots that there are significant differences in differential metabolites among the three types of gastric cancer. The top 50 heatmap reveals that the metabolic profiles of normal control tissues were more similar across the three types of GC, while the high‐abundance metabolites in tumor tissues of the three types of GC exhibited differences (Figure 1L).

### 3.3 Microbial Diversity In ZJU‐Typed Gastric Cancer

**Figure 2:**
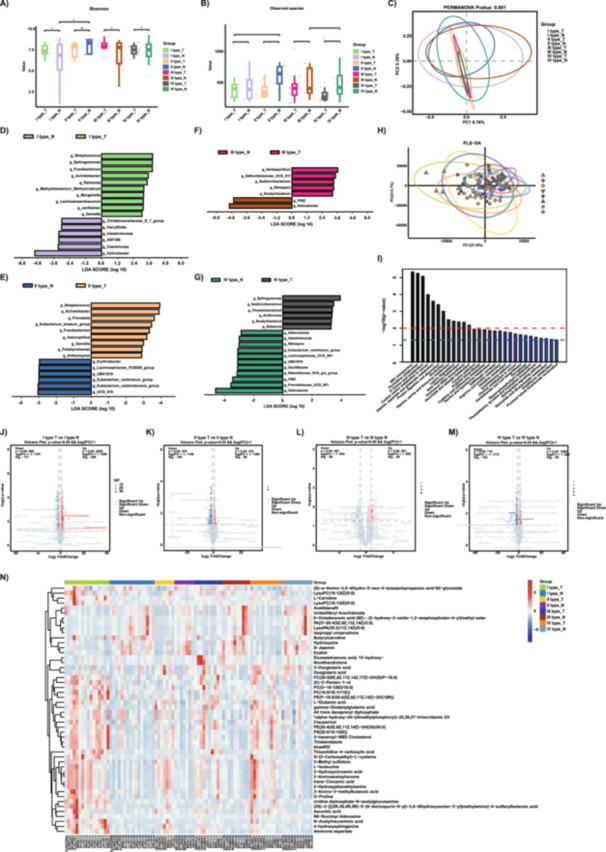
The differences in microbiota and metabolites among the three subtypes of ZJU classification. (A, B) The Shannon index and Observed species index for four subtypes of tumor tissues and their paired normal control tissues in the ZJU classification. (C) PCoA of weighted UniFrac distance demonstrated that the four subtypes of tumor tissues and matched nontumor tissues showed distinct clusters. (D–G) The LefSe analysis results reveal the biomarkers for the four subtypes of tumor tissues and the control tissues(LDA ＞ 3.0, *Q* ＜ 0.05). (H) PLS‐DA showed that three subtypes of tumor tissues and matched nontumor tissues were separated into eight clusters. (I) KEGG analysis indicates the significantly enriched metabolic pathways for differentially abundant metabolites. (J–M) The volcano plot illustrates the differentially abundant metabolites between the four subtypes (I, II, III, IV) of the ZJU classification and their respective control tissues (VIP > 1 and *p* value < 0.05). (N) Heatmap represents differential metabolites between eight groups. I type_T: I type GC tumor tissues; I type_N: I type GC normal tissues; II type_T: II type GC tumor tissues; II type_N: II type GC normal tissues; III type_T: III type GC tumor tissues; III type_N: III type GC normal tissues; IV type_T: IV type GC tumor tissues; IV type_N: IV type GC normal tissues; *p ≤ 0.05; **p ≤ 0.01; ***p ≤ 0.001.

Based on whole‐exome sequencing, our team conducted a subtyping analysis of GC patients in China. Ultimately, the patients were classified into four subtypes (I, II, III, and IV types), named the ZJU classification. Alpha diversity analysis revealed significant differences in Shannon index and Observed species among the eight groups (Figure 2A,B). We conducted pairwise comparisons of the ZJU classification and discovered that there was a significant difference in the Shannon index of normal tissue microbiota between the I‐type and II‐type. The tumor tissue microbiota of the I‐type showed significant differences in Observed species compared to the IV‐type. Additionally, there were significant differences in Observed species between the tumor tissues of the III‐type and IV‐type (Figure 2A,B). Beta diversity analysis indicated significant differences in confidence intervals among the eight groups (Figure 2C). Differential microbial findings between four subtypes of tumor tissues and normal control tissues were identified using LefSe analysis. At the genus level, tumor tissues of I‐type GC and normal control tissues exhibited differential microbes, including *Streptococcus*, *Sphingomonas*, *Fusobacterium*, *Acinetobacter*, and so on (Figure 2D). In contrast, the differential microbes in the II‐type GC primarily included *Streptococcus*, *Acinetobacte*r, *Prevotella*, and so on (Figure 2E). The differential microbes in the III‐type GC were mainly *Harbaspirillum*, *Sediminibacterium*, and *Defluviitaleaceae*_UCG_001 (Figure 2F). The IV‐type GC exhibited differential microbes such as *Sphingomonas*, *Sediminibacterium*, *Chryseobacterium*, and so on (Figure 2G). Among these four types of GC, the abundance of *Helicobacter pylori* (Hp) in all normal tissues was significantly higher than that in tumor tissues. The results were similar to those of the Lauren classification. When comparing the microbial differences between each individual classification's tumor and normal control tissue separately, the four classifications exhibited numerous common differential characteristics.

Further, we compared the microbial differences among four subtypes of tumors. The results indicated no significant difference in Shannon index but a significant difference in Observed species (Supporting Information S1: Figure S2A). PCoA analysis revealed no significant differences among the four subtypes (Supporting Information S1: Figure S2B). LefSe analysis identified microbial differences at the genus level, with notable taxa including MND1, *Prevotellaceae*_NK3B1_group, *Rhodoferax*, *Butyricicoccus*, *Prevotellaceae*_UCG_001, *Eubacterium_siraeum_group*, *Campylobacter*, *Actinomyces*, *Leptotrichia*, *Streptococcus*, *Fusobacterium*, *Haemophilus*, *Cloacibacterium*, *Lachnoanaerobaculum*, and *Gemella* among the four subtypes (Supporting Information S1: Figure S2C).

### 3.4 Distinct Metabolites Associated With the ZJU Classification of Gastric Cancer

In the ZJU classification, significant metabolic differences exist between tumor tissues of the four subtypes and normal control tissues as well. PLS analysis reveals differences in metabolites between the four subtypes in the ZJU classification (*Q*2 = 0.0195, *R*2 = −0.073) (Figure 2H). In Type I gastric cancer, there are 373 distinct metabolites between tumor and normal control tissues (Figure 2J). Type II gastric cancer tissues exhibit 588 differential metabolites compared to the control (Figure 2K). Type III gastric cancer has 292 differential metabolites, while Type IV gastric cancer has 237 differential metabolites compared to the control(VIP ≥ 1, log2FC ≥ 1 or ≤−1, *p* ≤ 0.05) (Figure 2L,M).

KEGG pathway analysis indicates that the differential metabolites in the four types are primarily associated with ABC transporters, Pyrimidine metabolism, D‐Amino acid metabolism, Central carbon metabolism in cancer, Alanine, aspartate and glutamate metabolism, Protein digestion and absorption, Alcoholism, Glycine, serine and threonine metabolism, Lysine degradation, and so on (Figure 2I). The heatmap reveals significant variations in the increased metabolites among the four subtypes (Figure 2N).

### 3.5 Comparison of the Lauren and ZJU Taxonomic Characteristics

The Lauren classification is a pathological typing, and the ZJU classification established by our research group previously has a certain correspondence with the Lauren classification (Table 1). The Sankey diagram shows that Type I is characterized by a higher prevalence of intestinal‐type gastric cancer, while Types III and IV are associated with mixed and diffuse types, respectively (Supporting Information S1: Figure S3). A comparison of the differential microbes between the Lauren and ZJU classifications reveals that at the genus level, both classifications include *Fusobacterium* and *Haemophilus* among their differential microbes (Supporting Information S1: Figures S1C,D and S2C). Comparing the top 50 differential metabolites between the Lauren and ZJU classifications reveals that 38 metabolites serve as differential indicators for both classifications. However, each classification also exhibits its own unique metabolic characteristics (Supporting Information S2: Table S1).

### 3.6 The Relationship Between Discriminative Genera and Metabolites in Different Classes

**Figure 3:**
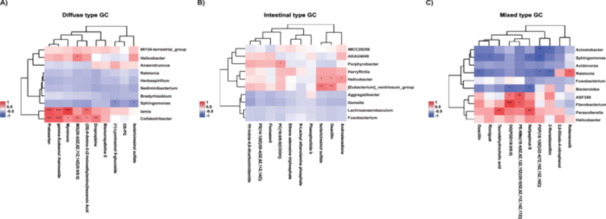
Differential microbiota‐metabolite correlation analysis among the three subtypes of Lauren classification. The association between the top 10 genera and 10 differential metabolites was analyzed using Pearson's correlation method. (A) Diffuse‐type GC tumor tissues VS normal tissues. (B) Intestinal‐type GC tumor tissues VS normal tissues. (C) Mixed‐type GC tumor tissues VS normal tissues. Red, positive correlations; Blue, negative correlations. A darker color indicates a more significant correlation. *p ≤ 0.05; **p ≤ 0.01; ***p ≤ 0.001.

The heatmap elucidates the interplay between the top 10 microbes exhibiting differential abundance and their associated metabolites across the Lauren subtypes of gastric cancer. Notably, in the normal control tissues of diffuse gastric cancer, a marked elevation in the abundance of Hp, *Lamprocystis*, and *Colidextribacter* was observed, which positively correlated with Pratosartan, gamma‐Eudesmol rhamnoside, Myrsinone, MG (20:4(5Z,8Z,11Z,14Z)/0:0/0:0), Dropropizine, and (2S)−6‐Amino‐2‐(2‐iminoethylamino) hexanoic Acid. Conversely, *Sphingomonas* was predominantly enriched in the diffuse‐type gastric cancer tissues, showing a significant negative correlation with (+)‐Lyoniresinol 9‐glucoside and Isolariciresinol sulfate (Figure 3A).

In the examination of associations between differential microbiota and metabolites in intestinal‐type gastric cancer, it was revealed that *Porphyrobacter*, *Harryflintia*, *Helicobacter*, and *Eubacterium_ventriosum_group*, exhibiting a pronounced increase in abundance in normal control tissues, were significantly positively correlated with PC (16:0/9:0(COOH)), Isolariciresinol sulfate, Oxacillin, and Androstenedione (Figure 3B).

Regarding mixed‐type gastric cancer, a significant upsurge in the abundance of *Acinetobacter*, *Sphingomonas*, *Ralstonia,* and *Bacteroides* was noted, which were significantly negatively correlated with Oxacillin, Riociguat, DG(PGD1/8:0/0:0), Nelipepimut‐S, PGP (18:1(9Z)/22:4(7Z,10Z,13Z,16Z)), and 2‐Norastaxanthin. Moreover, the heightened abundance of ASF356, *Flavobacterium*, and *Parasutterella* in the normal control tissue of mixed‐type gastric cancer was significantly positively correlated with PE‐NMe (18:4(6Z,9Z,12Z,15Z)/20:5(5Z,8Z,11Z,14Z,17Z)), Nelipepimut‐S, Taurodehydrocholic acid, and DG(PGD1/8:0/0:0) (Figure 3C).

**Figure 4:**
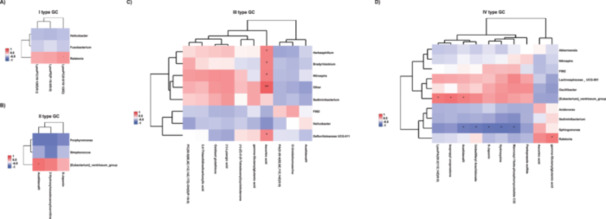
Differential microbiota‐metabolite correlation analysis among the four subtypes of ZJU classification. The association between the top 3 genera and three differential metabolites was analyzed using Pearson's correlation method. (A) I type GC tumor tissues VS normal tissues. (B) II type GC tumor tissues VS normal tissues. The association between the top 10 genera and 10 differential metabolites was analyzed using Pearson's correlation method. (C) III type GC tumor tissues VS normal tissues. (D) IV type GC tumor tissues VS normal tissues. *p ≤ 0.05; **p ≤ 0.01.

In the ZJU classification, there is no significant correlation between the top 10 differentially expressed metabolites and differential microbiota in Type I gastric cancer and Type II gastric cancer. Therefore, only the correlation analysis heatmap between the top 3 differentially expressed metabolites and differential microbiota was shown. The results indicate that in Type I gastric cancer, the significantly increased *Ralstonia* is significantly positively correlated with LysoPC(0:0/18:1(9Z)) (Figure 4A). In Type II gastric cancer, the significantly increased abundance of *Eubacterium_ventriosum_group* is significantly positively correlated with Acetildenafil in the control tissue (Figure 4B).

For the analysis of Type III GC and Type IV GC, the heatmap displays the correlation between the top 10 different metabolites and different microbiota. In Type III gastric cancer tissues, the significantly increased abundance of *Herbaspirillum*, *Bradyrhizobium*, *Nitrospira*, and *Defluviitaleaceae* UCG‐011 is significantly positively correlated with Ascorbic acid (Figure 4C). In contrast, in Type IV gastric cancer tissues, the increased abundance of *Sphingomonas* is significantly negatively correlated with Acetildenafil, Umbelliferyl Arachidonate, S‐Japonin, Hydroxyzine, and Mannosyl‐1beta‐phosphomycoketide C32. Additionally, the increased abundance of *Ralstonia* in gastric cancer tissues is significantly positively correlated with gamma‐Glutamyglutamic acid. In the normal control tissues, the increased abundance of *Eubacterium_ventriosum_group* is significantly positively correlated with Acetildenafil, isopropyl unoprostone, and LysoPA(20:2(11Z,14Z)/0:0) (Figure 4D).

### 3.7 Association between Differential Metabolites, Differential Microbiota, and Prognosis

**Figure 5:**
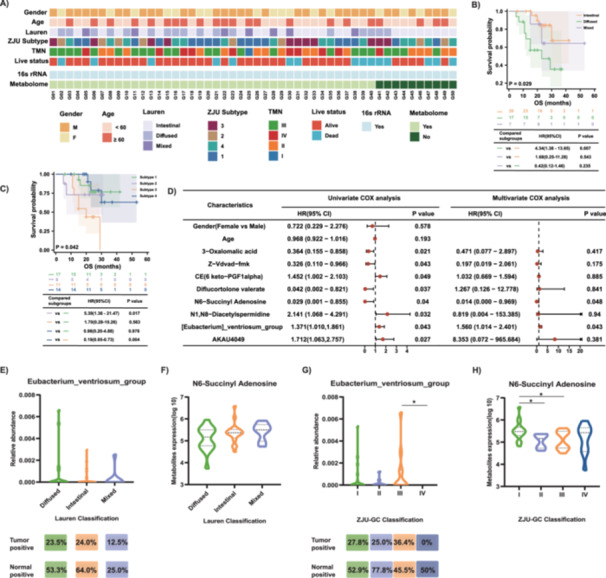
Clinical information and prognostic analysis of differential biomarkers in all gastric cancer patients. (A) The clinicopathological characteristics of patient cohort. (B) The relationship between the Lauren classification and survival. (C) The relationship between ZJU classification and survival. (D) Univariate and multivariate COX analysis of differential metabolites and differential microbiota. (E–H) The relative abundance of *Eubacterium_ventriosum_group* and N6‐Succinyl Adenosine in tumor tissues based on Lauren classification and ZJU classification.

Paired fresh‐frozen tumor tissue and adjacent normal tissue were collected from a cohort of 50 pathologically confirmed GC patients for 16S rRNA sequencing. Additionally, 40 of these pairs underwent nontargeted metabolomics sequencing. The clinicopathological data of the 50 patients are summarized in Table 1 and Figure 5A. Among the 50 gastric cancer patients, 33 (66.0%) were male, with a median age at the time of diagnosis of 60.5 years (range: 38–80 years). The majority of the patients 66.0%) were diagnosed at an advanced stage (Stage I/II: 17 patients, Stage III/IV: 33 patients). Besides, 54.0% of cases were identified as intestinal type and 34.0% were diffused type. According to the ZJU‐GC classification (Wang 2021a), there were 17, 8, 11, and 14 patients in types 1 through 4, respectively.

In terms of prognosis, it was observed that diffuse‐type patients had significantly worse outcomes compared to intestinal‐type patients [HR = 4.5468(1.394–14.829), ***p*** = 0.0120, Figure 5B]. Furthermore, within the ZJU classification, it was observed that subtype 3 had the worst prognosis, followed by subtype 2, while subtypes 1 and 4 displayed relatively more favorable prognosis (Figure 5C).

Through a comparative analysis between paired tumor and normal tissues, we have discerned 41 microbiota and 260 metabolites (|log2FC | > 1, *p* < 0.05, Supporting Information S2: Table S2). The differential analysis of microbiota and metabolites between tumor tissue and normal control tissue can be found in Supporting Information S1: Figure S4. Subsequently, prognostic analyses were conducted concerning these differential microbes and metabolites. In a univariate COX regression analysis, it was observed that several metabolites, including 3‐Oxalomalic acid, Z‐Vdvad‐fmk, CE (6 keto‐PGF1alpha), Diflucortolone valerate, N6‐Succinyl Adenosine, and N1, N8‐Diacetylspermidine, exhibited significant associations with prognosis. Similarly, certain microbial entities, *Eubacterium_ventriosum_group* and AKAU4049, displayed notable prognostic correlations. (*p* < 0.05, Supporting Information S2: Tables S2 and S3 and Figure 5D). Multivariate analysis showed that N6‐Succinyl Adenosine and *Eubacterium_ventriosum_group* were the independent prognostic factors (Figure 5D). Concurrently, the correlation analysis presented above also indicates that there is no significant association between N6‐Succinyl Adenosine and *Eubacterium_ventriosum_group*. The violin plots display the levels of N6‐Succinyl Adenosine and *Eubacterium_ventriosum_group* in tumor tissues categorized by Lauren classification and ZJU classification, respectively (Figure 5E‐H). *Eubacterium_ventriosum_group* positively correlates with poor prognosis, whereas N6‐Succinyl Adenosine negatively correlates with poor prognosis.

## 4 Discussion and Conclusion

Previous studies have described the differences in microbial composition in gastric cancer based on the Lauren classification (Liu et al. 2019). However, this study is the first to describe the differences in both microbial composition and metabolites between gastric cancer subtypes based on the Lauren and ZJU classifications. Additionally, we have conducted preliminary investigations into the microbial and metabolite differences that are associated with prognosis.

In the analysis of the Lauren classification, we found significant differences in microbiota and metabolites between diffuse‐type gastric cancer, intestinal‐type gastric cancer, and mixed‐type gastric cancer. In diffuse‐type gastric cancer, LefSe analysis revealed a significant increase in the abundance of *Sphingomonas*, *Prevotella*, *Sediminibacterium*, *Ralstonia*, *Herbaspirillum*, and *Bradyrhizobium* in tumor tissues. There is literature indicating an increased abundance of *Sphingomonas* in tissues and urine of different tumors, suggesting its association with tumor occurrence and development (Dai et al. 2021; Bukavina et al. 2023). However, previous studies have also shown that the abundance of *Sphingomonas* in the blood of gastric cancer patients is not higher than that of healthy controls (Dong et al. 2019). *Sphingomonas* is a common environmental microorganism and may contain the anti‐chlorine gene GlnRS (Miao et al. 2022). Their main function is the degradation of pollutants, and they may also be associated with the generation of inflammation (Chen et al. 2022). The correlation analysis between differential microbiota and differential metabolites revealed a significant negative correlation between *Sphingomonas* and Isolariciresinol sulfate as well as (+)‐Lyoniresinol 9‐glucoside. The abovementioned compounds belong to lignan compounds and possess antioxidant and anti‐inflammatory properties (Peron et al. 2023; Sampei et al. 2018). The observed negative correlation between *Sphingomonas* abundance and lignan metabolites likely stems from *Sphingomonas paucimobilis's* enzymatic degradation of lignan derivatives via the ligAB/ligC‐encoded pathway, converting substrates into 2‐pyrone‐4,6‐dicarboxylic acid (PDC) (Otsuka et al. 2006). This catabolic capacity directly reduces bioactive lignan pools, aligning with our dual‐omics findings of inverse microbial‐metabolite relationships.

In intestinal‐type gastric cancer, there is a significant increase in the abundance of *Fusobacterium*, *Eikenella*, *Acinetobacter*, *Alloprevotella*, *Bergeyella*, *Actinobacillus*, *Aggregatibacter*, *Rothia*, *Granulicatella*, *Lachnoanaerobaculum*, and *Gemella* in tumor tissues. The abovementioned microorganisms, most of which belong to oral microbiota, are associated with inflammation, cancer, and other conditions (Peng 2022a; Gao et al. 2018). Differential microbiota‐metabolite correlation analysis revealed that among the top 10 differential metabolites in intestinal‐type gastric cancer, none of them were significantly associated with the increased abundance of microorganisms in gastric cancer tissue. However, in the control tissues of intestinal‐type gastric cancer patients, the increased abundance of *Eubacterium_ventriosum_group* and Hp showed significant positive correlations with Isolariciresinol sulfate. *Porphyrobacter* showed a significant positive correlation with PC (16:0/9:0(COOH)). Previous studies have indicated a significant increase in *Porphyrobacte* in patients with bladder cancer recurrence, suggesting an association with poor prognosis (Wu et al. 2018). There are also studies indicating that *Porphyrobacter* can serve as a biomarker for polycystic ovary syndrome (PCOS) (Yang 2022b). Prior studies have established that lignans, including pinoresinol, isolariciresinol, and secoisolariciresinol, are generated through microbial biotransformation of natural plant‐derived lignan precursors by the intestinal microbiota. However, the specific bacterial taxa responsible for these metabolic conversions have not been fully characterized (Bannwart et al. 1989; Heinonen et al. 2001).

In mixed‐type gastric cancer tissues, the significantly increased microbial genera include *Acidovorax*, *Bacteroides*, *Sphingomonas*, *Ralstonia*, *Fusobacterium*, and *Acinetobacter*. Research has shown that *Acidovorax* exhibits significantly increased abundance in TP53‐mutated squamous cell lung cancer. This suggests a potential correlation between *Acidovorax* and TP53 mutations. However, the specific mechanisms by which *Acidovorax* may impact cancer are not yet clear (Greathouse et al. 2018). Correlation analysis has shown a significant positive association between increased abundance of *Parasutterella* and *Flavobacterium* in the normal control tissues of mixed‐type gastric cancer and compounds such as Taurodehydrocholic acid. Studies have suggested that *Parasutterella* may be involved in lipid metabolism and immune regulation (Henneke et al. 2022). On the other hand, *Flavobacterium* has been associated with colorectal cancer and also impacts the metabolism of flavonoids in the body (Speciani et al. 2022).

In the ZJU classification, compared to the normal control tissues of the four subtypes, *Mythylobacterium_Methylorubrum*, *Morganella*, *Lachnoanaerobaculum*, and *Janibacter* showed significantly increased abundance only in type I gastric cancer tissues. *Prevotella*, *Eubacterium_Siraeum*, *Porphyromonas*, and *Actinomyces* exhibited significantly increased abundance only in type II gastric cancer tissues. *Defluviitaleaceae* and *Nitrospira* showed significantly increased abundance only in type III gastric cancer tissues. *Chryseobacterium* and *Acidovorax* displayed significantly increased abundance only in type IV gastric cancer tissues. *Methylobacterium_Methylorubrum* primarily exists in environments such as soil, water, and plant surfaces (Xie et al. 2023; Anguita‐Maeso et al. 2022). Previous studies have found a significant increase in the abundance of *Methylobacterium_Methylorubrum* in body fluid and tissue samples from prostate cancer patients, as well as in distal gastric cancer tissue samples (Yang 2022a; Anguita‐Maeso et al. 2022). *Morganella* is a common gut microbiota, and studies have shown its association with a certain indole metabolite called indolimines in the intestine. This metabolite has been found to promote the development of colorectal cancer (Puschhof and Sears 2022). Previous studies have indicated that the abundance of *Lachnoanaerobaculum* is associated with the occurrence and development of oral cancer and lung cancer (Yang 2022c; Najafi et al. 2021). There is limited research on *Janibacter*, but early studies have suggested that it may be associated with bacteremia, a condition characterized by the presence of bacteria in the bloodstream (Loubinoux et al. 2005). *Eubacterium_Siraeum* has been suggested to be associated with chemotherapy resistance in lung cancer (Zhao et al. 2021). Multiple studies have indicated that *Porphyromonas* and *Actinomyces* are associated with tumor occurrence and development (Stasiewicz and Karpiński 2022; Tan et al. 2022; Lamont et al. 2022; Yachida et al. 2019; Zhu et al. 2018; Yacouba et al. 2022). Research has shown that *Porphyromonas* promotes cancer development by inducing cellular senescence through the secretion of bacterial metabolites, such as butyrate (Okumura et al. 2021). *Defluviitaleaceae* is associated with diseases such as rheumatoid arthritis, cardiac fibrosis, and Parkinson's disease (Bordoni et al. 2019; Shi et al. 2018; Du et al. 2022; Tong et al. 2019). Previous studies have indicated that the abundance of *Nitrospira* decreases during the progression from normal gastric tissue to gastritis and eventually to gastric cancer (Wang et al. 2020). However, in our observations of type III gastric cancer, there is a significant increase in the abundance of Nitrospira. Previous research has also suggested that patients with type III gastric cancer have an increased risk of peritoneal metastasis and poorer prognosis (Wang 2021a). These findings suggest a negative correlation between *Nitrospira* and the occurrence of gastric cancer while being positively associated with adverse prognosis in gastric cancer. Recent studies have also indicated that *Chryseobacterium* in tumor tissue of gastric cancer patients can serve as a distinguishing biomarker between gastric cancer and superficial gastritis, which is consistent with our findings of increased abundance of this microorganism in IV‐type gastric cancer tissue (Liu 2022a). Interestingly, the differential metabolite‐differential microbe correlation analysis revealed that the microorganisms significantly increased in abundance in III‐type gastric cancer tissues were positively correlated with Ascorbic acid (vitamin C). Over the past century, the idea of using vitamin C for cancer treatment has sparked considerable controversy. However, multiple studies have now indicated that vitamin C can be used for cancer therapy, enhancing the effectiveness of immunotherapy (Ngo et al. 2019; Peng 2022b). In III‐type gastric cancer, it is significantly correlated with increased abundance of harmful microorganisms, which may suggest a distinct metabolic profile in III‐type gastric cancer. Vitamin C may also have other harmful effects.

In our study on the ZJU classification, we found that III‐type and IV‐type gastric cancers tend to exhibit a histological pattern consistent with diffuse‐type gastric cancer, while I‐type gastric cancer leans toward intestinal‐type gastric cancer (Wang 2021a). Analyzing the results of different LefSe analyses, we identified three significantly increased microorganisms in III‐type gastric cancer tissues that were consistent with those found in diffuse‐type gastric cancer (*Herbaspirillum*, *Sediminibacterium*, *Bradyrhizobium*). Similarly, IV‐type gastric cancer showed four significantly increased microorganisms that were consistent with those found in diffuse‐type gastric cancer (*Sphingomonas*, *Sediminibacterium*, *Bradyrhizobium*, *Ralstonia*). On the other hand, I‐type gastric cancer tissues exhibited significantly increased abundance of four microorganisms that were consistent with those found in intestinal‐type gastric cancer (*Fusobacterium*, *Acinetobacter*, *Lachnoanaerobaculum*, and *Gemella*). These findings from a microbial perspective validate the relationship between the ZJU classification and the Lauren classification.

The KEGG analysis revealed that differential metabolites in the three subtypes of Lauren classification were mainly enriched in pathways such as the TCA cycle, Autophagy, and Glycolysis/Gluconeogenesis. On the other hand, differential metabolites in the four subtypes of ZJU classification were primarily associated with various metabolic pathways, including ABC transporters, Pyrimidine metabolism, d‐Amino acid metabolism, and so on. Volcano plots and heatmap analysis demonstrated distinct differential metabolites between the subtypes of Lauren classification and ZJU classification. In diffuse‐type tumor tissues, higher levels of 3,5‐di‐O‐(beta‐Glucopyranosyl) pelargonidin 6"‐O‐4, 6"‘‐O‐1‐cyclic malate, D‐Erythroascorbic acid 1′‐α‐d‐xylopyranoside, Androstenedione, 2,3‐Dihydro‐5H‐oxazolo(3,2‐a)thieno(3,2‐d)pyrimidin‐5‐one, 3alpha,7alpha,12alpha,16alpha‐tetrahydroxy‐5beta‐cholan‐24‐oic Acid, 5’,5”‘‐diadenosine triphosphate, and PGP(20:4(6E,8Z,11Z,13E)−2OH(5S,15S)/18:3(6Z,9Z,12Z)) were observed, while higher levels of 3Z,6Z,9Z−Heptadecatriene, Aglepristone, Lyoniresinol 9’−sulfate, Benzyl 6 − O−beta−D−apiofuranosyl−beta−D−glucoside, Phosphoribose, and 6−Heptenyl glucosinolate were found in intestinal‐type gastric cancer tissues. 3,5‐di‐O‐(beta‐Glucopyranosyl) pelargonidin 6"‐O‐4, 6"‘‐O‐1‐cyclic malate is a complex compound and a glycoside derivative of anthocyanin (Nakayama et al. 2000). D‐Erythroascorbic acid 1’‐α‐d‐xylopyranoside is a derivative of vitamin C. 3alpha,7alpha,12alpha,16alpha‐tetrahydroxy‐5beta‐cholan‐24‐oic Acid is a bile acid derivative, 5’,5"‘‐diadenosine triphosphate is a nucleotide, and PGP(20:4(6E,8Z,11Z,13E)−2OH(5S,15S)/18:3(6Z,9Z,12Z)) is a phospholipid. The detailed mechanisms of action of these mentioned metabolites on tumors have not been fully elucidated. Androstenedione is a sex hormone, and its imbalance may be associated with cancers such as pancreatic cancer and breast cancer (Fernández‐del Castillo et al. 1990; Chatterton 2022). Lyoniresinol 9’‐sulfate, 6‐Heptenyl glucosinolate, and Benzyl 6‐O‐beta‐d‐apiofuranosyl‐beta‐d‐glucoside are all derived from plants and have anti‐inflammatory effects (Saleem et al. 2021; Chiang et al. 2021). 3Z,6Z,9Z‐Heptadecatriene is a fatty acid, and Phosphoribose is a ribose phosphate that is an important component of nucleic acids and coenzymes (Twidle et al. 2017; Daniels et al. 2020). In the ZJU classification, we found that the content of (S)‐a‐Amino‐2,5‐dihydro‐5‐oxo‐4‐isoxazolepropanoic acid N2‐glucoside, L‐Carnitine, LysoPC(18:1(9Z)/0:0), and LysoPC(16:1(9Z)/0:0) was higher in type I gastric cancer tissues compared to the other three types. Additionally, there was an increase in the content of Eicosatetraenoic acid and Norethandrolone in type III gastric cancer. No relevant literature was found for (S)‐a‐Amino‐2,5‐dihydro‐5‐oxo‐4‐isoxazolepropanoic acid N2‐glucoside among the mentioned metabolites. L‐Carnitine is an amino acid derivative and is associated with cancer risk (Liu 2022b). LysoPC(18:1(9Z)/0:0) and LysoPC(16:1(9Z)/0:0) are two subtypes of phosphatidylcholine. Currently, there is no available research reporting their specific functions. Previous studies have indicated an increased level of Eicosatetraenoic acid (ETA) in colorectal cancer, and it plays a crucial role in cardiovascular biology, carcinogenesis, and various inflammatory diseases (Neoptolemos et al. 1991; Wang 2021b). Norethandrolone is a hormone analog, and studies have shown that it can improve the survival rate of elderly patients with acute myeloid leukemia (Pigneux et al. 2017; Henderson et al. 1973).

We further explored the associations and differences between the Lauren classification and the ZJU classification. It was found that both classifications share differential microbiota, including *Fusobacterium* and *Haemophilus*. Additionally, 38 of the top 50 differential metabolites are common to both classifications. However, distinct classifications also exhibit unique differential microbiota and metabolite characteristics.

To analyze whether differential metabolites and differential microbiota between tumor tissues and control tissues are associated with prognosis, we first compared T and N and identified 260 significantly different metabolites and 41 different microbial taxa at the genus level. In this small‐scale study, we found that patients with diffuse‐type gastric cancer had worse survival compared to those with mixed‐type and intestinal‐type gastric cancer, which is consistent with previous large‐scale studies (Chen et al. 2016; Pernot et al. 2020). The ZJU classification indicated that types II and III had a poorer prognosis, consistent with previous findings (Wang 2021a). Multivariate survival analysis revealed a negative correlation between N6‐Succinyl Adenosine and patient prognosis and a significant positive correlation between *Eubacterium_ventriosum_group* and patient prognosis.

Comparing the relative abundance of *Eubacterium_ventriosum_group* in the three subtypes (diffuse‐type, mixed‐type, and intestinal‐type) of T tissues according to the Lauren classification, we found that diffuse‐type gastric cancer had a higher abundance of *Eubacterium_ventriosum_group* compared to intestinal‐type gastric cancer, but the difference was not significant. We also examined the positivity rate of *Eubacterium_ventriosum_group* in tumor tissues and found that the positivity rate was lowest in mixed‐type gastric cancer, while it was comparable in intestinal‐type and diffuse‐type gastric cancer tissues. Similarly, the differential metabolite N6‐Succinyl Adenosine showed a higher abundance in intestinal‐type gastric cancer compared to mixed‐type gastric cancer, but the difference was not significant. The trends of these differential markers were consistent with the results of the prognostic analysis.

In the ZJU classification, we observed that type III gastric cancer had the highest relative abundance of *Eubacterium_ventriosum_group*, with a positivity rate of approximately 36%, while in type IV gastric cancer tissues, the positivity rate of this microorganism was 0%. Our previous research indicated that type III and type IV gastric cancers are similar in terms of genomic stability, pathological classification, and metastatic tendencies. However, type IV gastric cancer has a very good prognosis and is associated with mutations in several genes (Wang 2021a). Based on this study, we speculate that this microorganism may be negatively correlated with the mutational characteristics of tumor tissues. Overall, the comprehensive analysis suggests that the metabolites and microbiota associated with the ZJU classification may be more relevant to prognosis.

In summary, we have identified microbial and metabolic differences among different subtypes based on the Lauren classification and ZJU classification. We conducted a survival analysis on the differential microbiota and metabolites. Additionally, we discussed the correlation between differential metabolites, microbiota, and prognosis in different subtypes. This study further characterizes the microbial and metabolic features of the Lauren and ZJU subtypes, providing a foundation for exploring the mechanisms underlying the development and progression of different gastric cancer subtypes.

## 5 Limitations of the Study

Our study identified microbiota and metabolite differences between Lauren and ZJU classifications, but it has limitations. The sample size was small, potentially biasing results and limiting their broader applicability. Metabolite profiling showed some drug molecules, influenced by patients' comorbidities and medication use. Nontargeted metabolomics is variable, and the lack of a validation set to confirm findings is another constraint.

## Author Contributions

**Yan Yang:** conceptualization; writing – review and editing; writing – original draft; methodology; funding acquisition. **Liping Wen:** methodology; data curation; visualization; formal analysis. **Wu Lin:** visualization; formal analysis; data curation. **Yiran Chen:** formal analysis; methodology; data curation; visualization. **Rui Yang:** visualization; formal analysis. **Chao He:** methodology; resources; data curation; formal analysis. **Yingzi Zhang:** visualization. **Jing Zhang:** data curation; resources. **Haohao Wang:** resources; data curation; formal analysis. **Haiyong Wang:** methodology; data curation; resources; writing – review and editing. **Lisong Teng:** writing – review and editing; funding acquisition; resources.

## Ethics Statement

The research was approved by the Ethics Committee of the First Affiliated Hospital, School of Medicine, Zhejiang University (IIT20230798A). Informed written consent was obtained from each patient before enrollment.

## Conflicts of Interest

None declared.

## Supporting information

Figure S1.

Figure S2.

Figure S3.

Figure S4.

Supporting Figure Legends.

Supporting Tables.

## Data Availability

Sequence data that support the findings of this study have been deposited in the National Center of Biotechnology Information with the primary accession code PRJNA1170490. The project is publicly accessible at: https://www.ncbi.nlm.nih.gov/bioproject/PRJNA1170490 (Last accessed: 2025‐08‐18).
