# Supplementary figures and images for: Microbial and Metabolomic Variations Correlated With Gastric Cancer Subtypes and Prognosis
Source: Microbiologyopen. 2025 Nov 10;14(6):e70139. doi: 10.1002/mbo3.70139 (PMC12598303; doi:10.1002/mbo3.70139)

A)

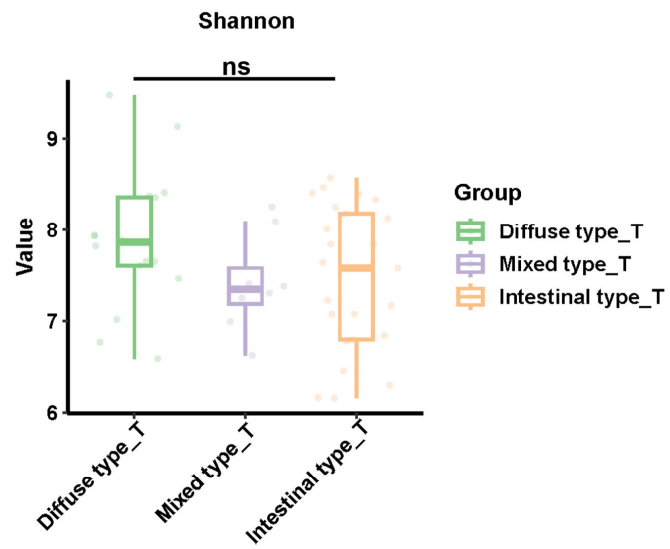

Observed species

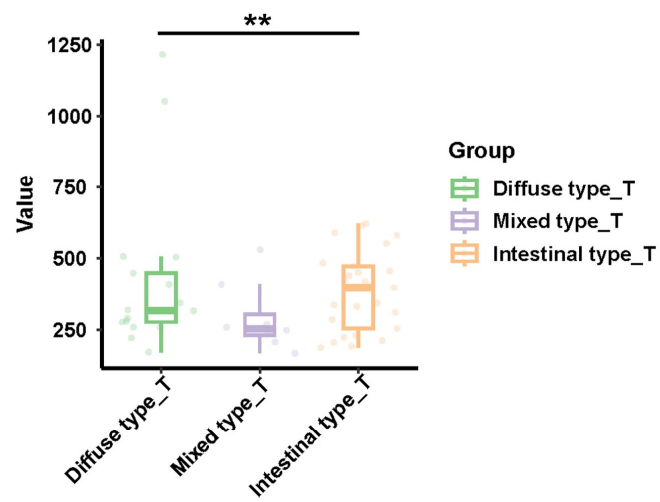

B)

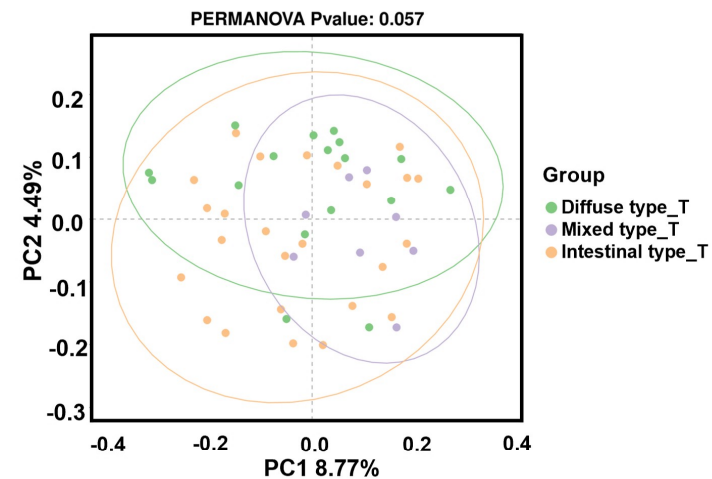

C)

Cladogram

Diffuse type\_T

Mixed type\_T

Intestinal type\_T

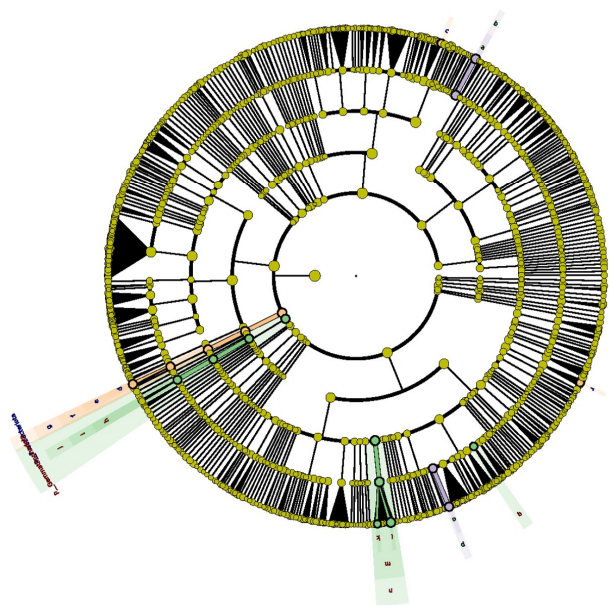

D)

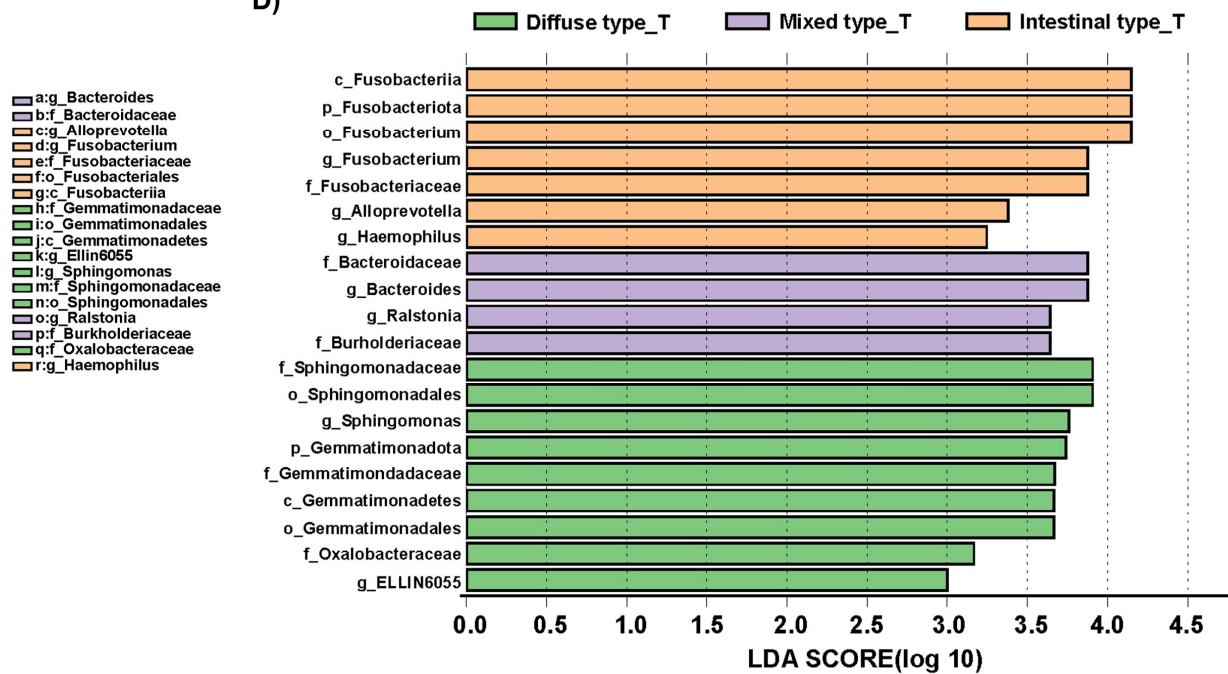

Supplement: Supplementary file 1 — Figure S1. [file MBO3-14-e70139-s001.pdf]

**C)**

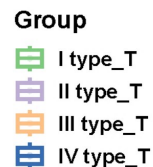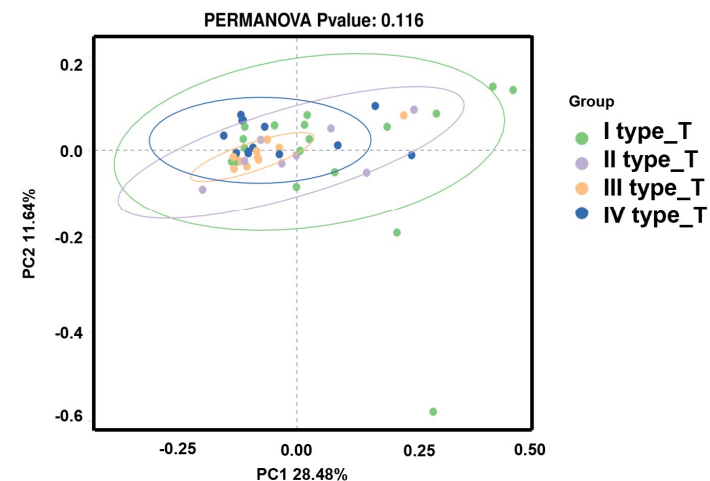

**C)**

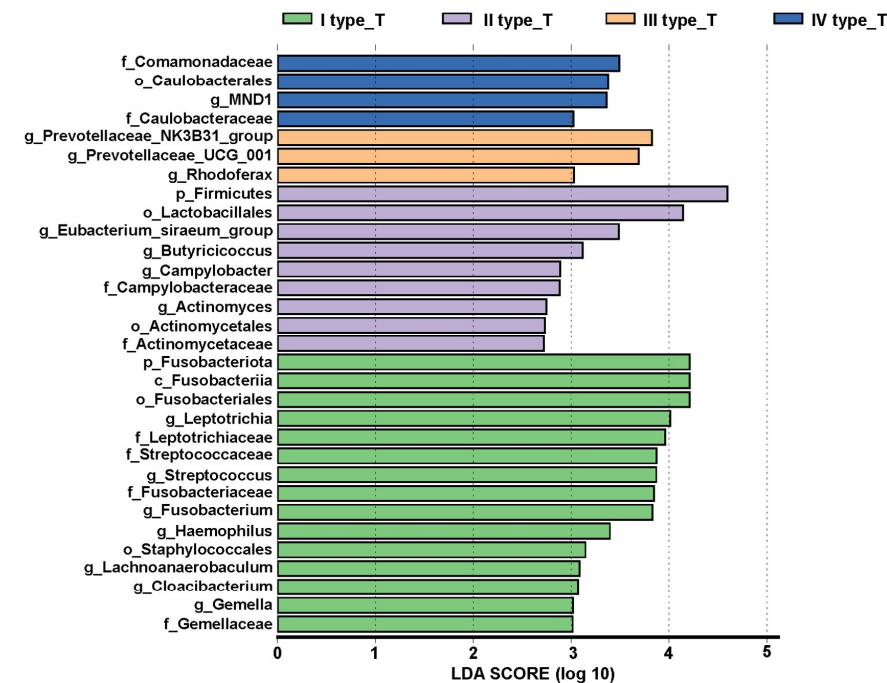

Supplement: Supplementary file 2 — Figure S2. [file MBO3-14-e70139-s003.pdf]

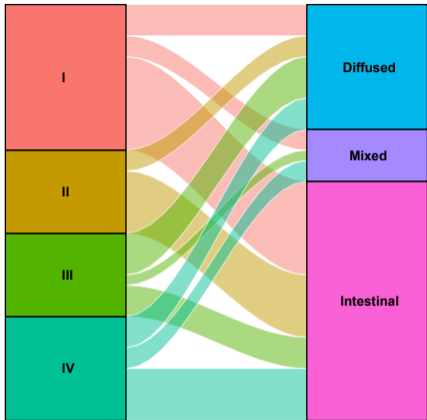

**ZJU classification**

**Lauren classification**

Supplement: Supplementary file 3 — Figure S3. [file MBO3-14-e70139-s006.pdf]

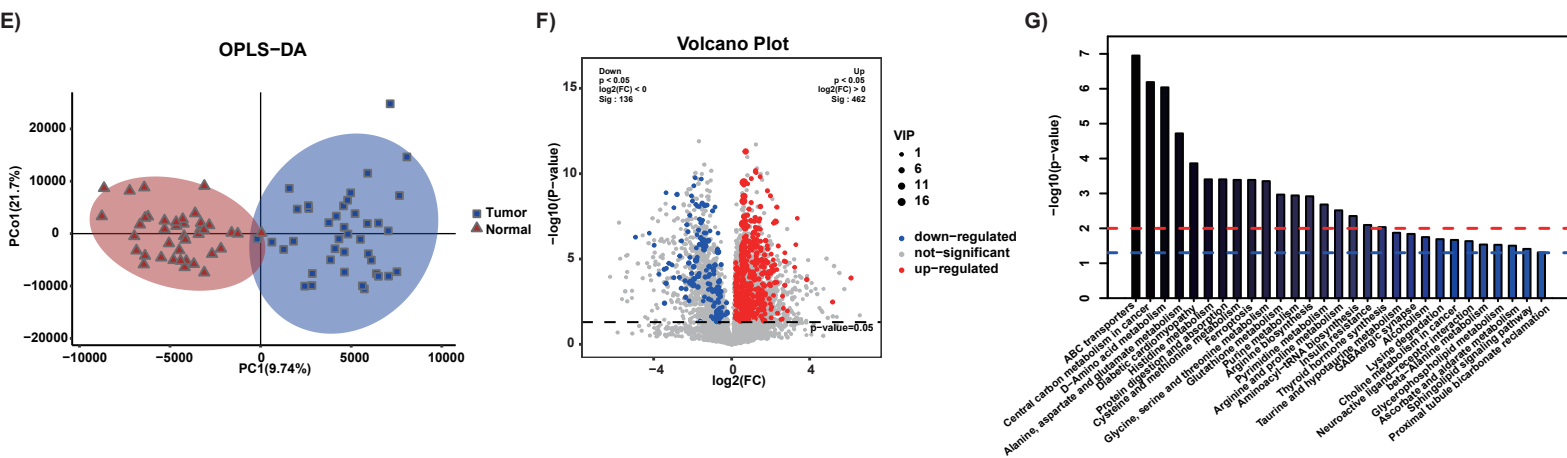

Supplement: Supplementary file 4 — Figure S4. [file MBO3-14-e70139-s002.pdf]
