# Supplementary material for: Microbial and Metabolomic Variations Correlated With Gastric Cancer Subtypes and Prognosis
Source: Microbiologyopen. 2025 Nov 10;14(6):e70139. doi: 10.1002/mbo3.70139 (PMC12598303; doi:10.1002/mbo3.70139)
Supplement: Supplementary file 5 — Supporting Figure Legends. [file MBO3-14-e70139-s005.docx]

**Supplementary Figure Legends**

**Fig.S1** **Differential microbiota in tumor tissues of Diffuse type GC, Intestinal type GC and Mixed type GC.** A) The Shannon index and Observed species index of microbiota among the three subtypes of tumor tissues in Lauren classification. **: p≤0.01. B) PCoA of weighted UniFrac distance demonstrated that the three subtypes tumor tissues showed distinct clusters (p=0.057). C, D) Differential taxa and cladogram identified by LefSe analysis (LDA＞3.0, Q＜0.05). Diffuse type_T: Diffuse type GC tumor tissues; Intestinal type_T: Intestinal type GC tumor tissues; Mixed type_T: Mixed type GC tumor tissues.

**Fig.S2** **Differential microbiota in tumor tissues of I type GC, II type GC, III type GC and IV type GC.** A) The Shannon index and Observed species index of microbiota among the four subtypes of tumor tissues in ZJU classification. *: p≤0.05. B) PCoA of weighted UniFrac distance demonstrated that the four subtypes tumor tissues showed distinct clusters (p=0.116). C, D) Differential taxa and cladogram identified by LefSe analysis (LDA＞3.0, Q＜0.05). I type_T: I type GC tumor tissues; II type_T: II type GC tumor tissues; III type_T: III type GC tumor tissues; IV type_T: IV type GC tumor tissues.

**Fig.S3** **Sankey Diagram of Correspondence between ZJU Classification and Lauren Classification.**

**Fig.S4 The differential microbiota and differential metabolites between gastric cancer tumor tissues and normal control tissues.** A, B) The Shannon index and Observed species index of microbiota in gastric cancer tumor tissues and control tissues. C) PCoA of weighted UniFrac distance demonstrated that tumor tissues and normal tissues showed distinct clusters (p=0.001). D) LefSe analysis reveals microbial biomarkers for tumor tissues and control tissues (LDA＞3.0, Q＜0.05). E) OPLS-DA showed that Tumor and Normal were separated into two clusters. F) Volcano map of different metabolites between Distal T and Distal N. (VIP > 1, p value < 0.05, |log2FC|＞1). G) KEGG pathway analysis indicates enriched pathways for differential metabolites. H) Heatmap representative differentially metabolites between Tumor and Normal. *: p≤0.05.
