# Supplementary material for: Microbial and Metabolomic Variations Correlated With Gastric Cancer Subtypes and Prognosis
Source: Microbiologyopen. 2025 Nov 10;14(6):e70139. doi: 10.1002/mbo3.70139 (PMC12598303; doi:10.1002/mbo3.70139)
Supplement: Supplementary file 6 — Supporting Tables. [file MBO3-14-e70139-s004.docx]

**Table S1.** **The top 50 differential metabolites between the Lauren and ZJU classifications.**

| **Top50 different metabolites of Lauren classification** | **Top50 different metabolites of ZJU classification** |
| --- | --- |
| PE-NMe2(15:0/22:5(4Z,7Z,10Z,13Z,16Z)) | LysoPC(18:1(9Z)/0:0) |
| L-Isoleucine | PC(16:0/18:1(11E)) |
| trans-Cinnamic acid | L-Isoleucine |
| PC(16:0/18:1(11E)) | trans-Cinnamic acid |
| 2-Hydroxycinnamic acid | PC(20:5(6E,8Z,11Z,14Z,17Z)-OH(5)/P-16:0) |
| PC(20:5(6E,8Z,11Z,14Z,17Z)-OH(5)/P-16:0) | 2-Hydroxycinnamic acid |
| S-Japonin | S-Japonin |
| 9-Octadecenoic acid (9Z)-, (2-hydroxy-2-oxido-1,2-oxaphospholan-4-yl)methyl ester | L-Carnitine |
| 2-Hydroxyphenethylamine | 9-Octadecenoic acid (9Z)-, (2-hydroxy-2-oxido-1,2-oxaphospholan-4-yl)methyl ester |
| L-Carnitine | Acetildenafil |
| Thiabendazole | 2-Hydroxyphenethylamine |
| Acetildenafil | Thiabendazole |
| bhas#32 | 3-Amino-3-methylbutanoic acid |
| 3-Amino-3-methylbutanoic acid | 3-Methyl sulfolene |
| Ecabet | bhas#32 |
| Indoleacrylic acid | Ecabet |
| 3-Methyl sulfolene | D-Proline |
| 3-hexanoyl-NBD Cholesterol | 3-hexanoyl-NBD Cholesterol |
| D-Proline | PA(O-20:4(5Z,8Z,11Z,14Z)/2:0) |
| Thiazolidine-4-carboxylic acid | LysoPA(20:2(11Z,14Z)/0:0) |
| PA(O-20:4(5Z,8Z,11Z,14Z)/2:0) | PS(20:0/16:1(9Z)) |
| All trans decaprenyl diphosphate | Eicosatetraenoic acid, 15-hydroxy- |
| PS(20:0/16:1(9Z)) | L-Glutamic acid |
| LysoPA(20:2(11Z,14Z)/0:0) | 4-hydroxysphinganine |
| L-Glutamic acid | Hydroxyzine |
| 1alpha-hydroxy-24-(dimethylphosphoryl)-25,26,27-trinorvitamin D3 / 1alpha-hydroxy-24-(dimethylphosphoryl)-25,26,27-trinorcholecalciferol | LysoPC(16:1(9Z)/0:0) |
| 4-hydroxysphinganine | Ascorbic acid |
| LysoPC(16:1(9Z)/0:0) | Thiazolidine-4-carboxylic acid |
| Ascorbic acid | Uridine diphosphate-N-acetylglucosamine |
| PC(O-14:0/18:0) | (S)-a-Amino-2,5-dihydro-5-oxo-4-isoxazolepropanoic acid N2-glucoside |
| Hydroxyzine | N-Acetylneuraminic acid |
| Uridine diphosphate-N-acetylglucosamine | All trans decaprenyl diphosphate |
| isopropyl unoprostone | Ammonia aspartate |
| (S)-Laudanosine | 3-Oxoglutaric acid |
| PE(20:4(5Z,8Z,11Z,14Z)-OH(20)/20:0) | Clausarinol |
| 2-Aminoacetophenone | N6-Succinyl Adenosine |
| PC(O-18:1(9Z)/16:0) | isopropyl unoprostone |
| Diisopropanolamine | Norethandrolone |
| Clausarinol | S-(2-Carboxyethyl)-L-cysteine |
| 5S-HETE di-endoperoxide | PC(O-18:1(9Z)/16:0) |
| Ammonia aspartate | 2-Aminoacetophenone |
| 2-Heptanethiol | gamma-Glutamylglutamic acid |
| PC(PGF1alpha/16:0) | 1alpha-hydroxy-24-(dimethylphosphoryl)-25,26,27-trinorvitamin D3 / 1alpha-hydroxy-24-(dimethylphosphoryl)-25,26,27-trinorcholecalciferol |
| PE(20:3(6,8,11)-OH(5)/P-18:0) | (E)-2-Penten-1-ol |
| (E)-2-Penten-1-ol | Umbelliferyl Arachidonate |
| V-Pyrro/NO | PE(20:4(5Z,8Z,11Z,14Z)-OH(20)/20:0) |
| (2S)-2-[[(2R,3S,4R,5R)-5-(6-Aminopurin-9-yl)-3,4-dihydroxyoxolan-2-yl]methylamino]-4-sulfanylbutanoic acid | Oxoglutaric acid |
| D-Glutamine | (2S)-2-[[(2R,3S,4R,5R)-5-(6-Aminopurin-9-yl)-3,4-dihydroxyoxolan-2-yl]methylamino]-4-sulfanylbutanoic acid |
| Hexanoylcarnitine | PE(P-18:0/20:4(5Z,8Z,11Z,14Z)-OH(18R)) |
| Umbelliferyl Arachidonate | Butyrylcarnitine |

**Table S2. Univariate Cox regression analysis for the association between altered metabolites and OS.**

| **Variable** | **HR** | **Lower_CI** | **Upper_CI** | **P_Value** |
| --- | --- | --- | --- | --- |
| Semilepidinoside B | 1.612091 | 0.867726 | 2.994995 | 0.130782 |
| Adenosine | 0.776325 | 0.368547 | 1.635286 | 0.505359 |
| Sulfinpyrazone sulfide | 1.626461 | 0.876545 | 3.017958 | 0.123029 |
| alpha-D-xylose 1-phosphate | 1.029451 | 0.485193 | 2.184224 | 0.939716 |
| Evogliptin | 0.96722 | 0.500477 | 1.869244 | 0.921021 |
| Isoproterenol | 0.707577 | 0.324541 | 1.542686 | 0.384398 |
| 5-Fluoro-8-hydroxy-2-(dipropylamino)tetralin | 0.905761 | 0.446245 | 1.838461 | 0.784053 |
| Moxifloxacin | 1.119327 | 0.588184 | 2.130104 | 0.731317 |
| **3-Oxalomalic acid** | 0.364321 | 0.15469 | 0.858037 | 0.020873 |
| 7,8-Dihydrovomifoliol 9-[rhamnosyl-(1->6)-glucoside] | 1.248929 | 0.773048 | 2.017759 | 0.363762 |
| Naloxegol | 1.296812 | 0.80338 | 2.093309 | 0.287395 |
| **Z-Vdvad-fmk** | 0.325941 | 0.109923 | 0.966477 | 0.043233 |
| (1,10,11,12,14,23-Hexahydroxy-6,10,19-trimethyl-24-oxa-4-azaheptacyclo[12.12.0.02,11.04,9.015,25.018,23.019,25]hexacosan-22-yl) 3,4-dimethoxybenzoate | 1.539516 | 0.946848 | 2.503159 | 0.081905 |
| Mycotoxin T 2 | 1.030595 | 0.602544 | 1.762737 | 0.912371 |
| 5-(Galactosylhydroxy)-L-Lysine | 1.50576 | 0.839901 | 2.699501 | 0.169383 |
| Canarigenin 3-[glucosyl-(1->4)-6-deoxy-alloside] | 1.102795 | 0.619114 | 1.96435 | 0.739745 |
| Longamide | 1.174335 | 0.701105 | 1.966983 | 0.541435 |
| 14,15-LTE4 | 0.948296 | 0.492396 | 1.826306 | 0.873855 |
| STROPHANTHIDIN SEMICARBAZIDE | 1.126695 | 0.598974 | 2.119359 | 0.711353 |
| DG(20:5(6E,8Z,11Z,14Z,17Z)-OH(5)/i-20:0/0:0) | 1.348328 | 0.82266 | 2.20989 | 0.235791 |
| Neryl rhamnosyl-glucoside | 1.129778 | 0.627258 | 2.034883 | 0.684419 |
| Vidarabine | 0.645563 | 0.224535 | 1.856069 | 0.416685 |
| 2-(Methylthiomethyl)-3-phenyl-2-propenal | 0.935608 | 0.440072 | 1.989134 | 0.862686 |
| Linderachalcone | 1.073699 | 0.556602 | 2.071191 | 0.832005 |
| N-Arachidonoyl Tryptophan | 0.993619 | 0.479521 | 2.058884 | 0.986259 |
| Trigonelline | 0.758872 | 0.369956 | 1.556636 | 0.451612 |
| 26:5(8Z,11Z,14Z,17Z,20Z) | 0.860063 | 0.439613 | 1.682635 | 0.659748 |
| Nopalinic acid | 0.540705 | 0.226001 | 1.293631 | 0.16712 |
| (2S)-2-Amino-6-[(3-formylpiperidin-1-yl)amino]hexanoic acid | 1.217618 | 0.711079 | 2.084991 | 0.473078 |
| 14,15-LTD4 | 1.106747 | 0.581312 | 2.107111 | 0.757526 |
| Avenin | 0.846339 | 0.432737 | 1.655255 | 0.625925 |
| 1,7-Dimethylguanosine | 0.711364 | 0.291346 | 1.7369 | 0.454603 |
| (2S)-2-[[(2R,3S,4R,5R)-5-(6-Aminopurin-9-yl)-3,4-dihydroxyoxolan-2-yl]methylamino]-4-sulfanylbutanoic acid | 0.807745 | 0.355605 | 1.83477 | 0.610008 |
| PA(20:3(8Z,11Z,14Z)-2OH(5,6)/2:0) | 1.088812 | 0.649407 | 1.825531 | 0.746919 |
| (2R,3R)-2-Aminooctadecane-1,3-diol | 0.921182 | 0.413011 | 2.054609 | 0.84102 |
| Marbofloxacin | 0.759178 | 0.338856 | 1.700871 | 0.503216 |
| Murrayanol | 1.196761 | 0.665631 | 2.151695 | 0.548435 |
| Tenovin-6 | 0.978622 | 0.537068 | 1.783204 | 0.943726 |
| 4'-Azidocytidine | 1.175604 | 0.630064 | 2.1935 | 0.611183 |
| N-Acetyl-D-glucosamine | 0.777868 | 0.38025 | 1.591267 | 0.491524 |
| Glutamylaspartic acid | 0.531642 | 0.174621 | 1.618608 | 0.26605 |
| 4-Amino-1-[(2R,5R)-5-(aminomethyl)-3,4-dihydroxyoxolan-2-yl]pyrimidin-2-one | 0.983327 | 0.465777 | 2.075952 | 0.964823 |
| PGP(i-12:0/i-12:0) | 1.341578 | 0.794968 | 2.264031 | 0.271083 |
| 2-[4-(Carboxymethyl)-1,4,8,11-tetrazabicyclo[6.6.2]hexadecan-11-yl]acetic acid | 0.697776 | 0.31991 | 1.521963 | 0.365781 |
| Tyrosyl-Glutamate | 0.684017 | 0.289912 | 1.613862 | 0.385874 |
| Testosterone phenylpropionate | 1.430074 | 0.843776 | 2.423761 | 0.183875 |
| Garbanzol | 1.299161 | 0.556853 | 3.030995 | 0.54485 |
| glas#26 | 1.272085 | 0.680201 | 2.379004 | 0.451178 |
| 20-Cooh ltb4 | 1.259918 | 0.730571 | 2.172812 | 0.406007 |
| omega-linoleoyloxy-Cer(d19:1/32:0) | 0.933021 | 0.494901 | 1.758994 | 0.830316 |
| Cerulenin | 0.533401 | 0.16834 | 1.690134 | 0.285486 |
| Diphenoxylate | 1.379631 | 0.851018 | 2.236594 | 0.191715 |
| Acetylcysteine | 0.482523 | 0.207521 | 1.121956 | 0.090518 |
| Aspacoside E | 1.387317 | 0.905038 | 2.126595 | 0.133063 |
| LysoPC(22:4(7Z,10Z,13Z,16Z)/0:0) | 1.10214 | 0.572095 | 2.123274 | 0.77128 |
| PE(20:4(5Z,8Z,11Z,14Z)-OH(20)/20:4(5Z,8Z,11Z,14Z)) | 1.29012 | 0.756892 | 2.199006 | 0.349146 |
| Fluocortin | 1.098081 | 0.597107 | 2.019373 | 0.763408 |
| FAPy-adenine | 1.441612 | 0.773057 | 2.688345 | 0.249984 |
| 1-Methyladenosine | 0.635067 | 0.240798 | 1.674893 | 0.358825 |
| Hydroxyzine | 1.05561 | 0.521223 | 2.137881 | 0.880522 |
| N1-Acetylspermidine | 0.456346 | 0.155405 | 1.340057 | 0.15347 |
| Acetildenafil | 1.059001 | 0.608968 | 1.841611 | 0.839088 |
| PC(16:1(9Z)/LTE4) | 1.264764 | 0.694062 | 2.304734 | 0.442976 |
| L-Asparagine | 0.461258 | 0.133183 | 1.597491 | 0.222132 |
| Serylglutamic acid | 1.107536 | 0.52198 | 2.349967 | 0.790154 |
| 5,9:7,10a-Dimethano-10aH-[1,3]dioxocino[6,5-d]pyrimidine-4,7,10,11,12-pentol, octahydro-12-(hydroxymethyl)-2-imino-,(4R,4aR,5R,7S,9S,10S,10aR,11S,12S)- | 0.922294 | 0.459448 | 1.851407 | 0.82002 |
| Ethyl 3-mercaptobutyrate | 0.589738 | 0.14409 | 2.413703 | 0.462676 |
| 1,6-anhydro-N-acetyl-beta-muramate | 1.051573 | 0.620722 | 1.781482 | 0.851688 |
| Fusarin C | 0.409227 | 0.096037 | 1.743776 | 0.227006 |
| Pyroglutamic acid | 0.512925 | 0.188581 | 1.395114 | 0.190963 |
| 3,4-Dimethyl-5-propyl-2-furanpentadecanoic acid | 0.748531 | 0.343041 | 1.633331 | 0.466883 |
| 8-Epiiridodial glucoside tetraacetate | 0.969557 | 0.456532 | 2.059089 | 0.935879 |
| N-Gluconyl ethanolamine phosphate | 0.633822 | 0.254733 | 1.577062 | 0.32687 |
| 3Z,6Z,9Z,12Z,15Z-Pentacosapentaene | 0.655608 | 0.255011 | 1.685506 | 0.380851 |
| 2-Keto-3-Deoxy-D-Mannooctanoic Acid | 0.485448 | 0.125131 | 1.883305 | 0.29612 |
| S-Japonin | 1.080326 | 0.551575 | 2.115949 | 0.821773 |
| Glutaminylaspartic acid | 0.719107 | 0.318357 | 1.624326 | 0.427691 |
| (1S)-1-(3-Methoxyphenyl)-2-[4-[4-[(propan-2-ylideneamino)oxymethyl]phenyl]triazol-1-yl]ethanol | 1.064509 | 0.544261 | 2.082054 | 0.855077 |
| Glutamylglycine | 0.424305 | 0.1138 | 1.582027 | 0.201673 |
| Rifamycin W | 1.403367 | 0.860159 | 2.289623 | 0.174838 |
| 4-hydroxysphinganine | 0.507028 | 0.160034 | 1.606397 | 0.248353 |
| Threonylglutamic acid | 0.97025 | 0.375597 | 2.506372 | 0.950266 |
| Mannosyl-1beta-phosphomycoketide C32 | 1.268504 | 0.697326 | 2.30753 | 0.435933 |
| (R)-beta-Aminoisobutyric acid | 0.369415 | 0.077477 | 1.76138 | 0.211443 |
| 7-Methyl-2'-deoxyguanosine-3'-monophosphate | 0.788321 | 0.336738 | 1.845498 | 0.583652 |
| Tetraphyllin B sulfate | 0.373746 | 0.095676 | 1.459994 | 0.156883 |
| 4-Trimethylammoniobutanoic acid | 0.335358 | 0.068581 | 1.639877 | 0.177282 |
| 3'-Azido-3'-deoxythymidine, 98% | 0.773985 | 0.285901 | 2.095314 | 0.614112 |
| 2-Deoxy-2,3-dehydro-n-acetyl-neuraminic acid | 0.716657 | 0.289695 | 1.772887 | 0.470964 |
| N-Isobutyryl-L-cysteine | 0.662456 | 0.28383 | 1.546167 | 0.340966 |
| 2-hydroxymethylolanzapine | 0.45158 | 0.093533 | 2.180245 | 0.322335 |
| GSK264220A | 0.797941 | 0.38484 | 1.654481 | 0.544056 |
| Sirolimus | 1.321111 | 0.771834 | 2.261282 | 0.30986 |
| Elaterinide | 1.289384 | 0.784805 | 2.118376 | 0.315687 |
| Symmetric dimethylarginine | 0.326664 | 0.077535 | 1.37628 | 0.127331 |
| 16&alpha;-hydroxyprednisolone | 0.487541 | 0.149507 | 1.589872 | 0.233588 |
| Thiazolidine-4-carboxylic acid | 1.266921 | 0.746219 | 2.150962 | 0.381012 |
| S-Cysteinosuccinic acid | 0.365278 | 0.094818 | 1.407201 | 0.14332 |
| LysoPA(20:2(11Z,14Z)/0:0) | 1.401664 | 0.793095 | 2.477207 | 0.245182 |
| 4-Keto-4'-hydroxyalloxanthin | 0.698861 | 0.286485 | 1.704822 | 0.430991 |
| Americine | 0.797869 | 0.400554 | 1.589287 | 0.520702 |
| Lysylvaline | 0.524577 | 0.173932 | 1.582122 | 0.252023 |
| Cer(t18:1(6OH)/31:0) | 0.74347 | 0.316117 | 1.748554 | 0.49692 |
| Aspartyl-Tryptophan | 0.76843 | 0.376345 | 1.568997 | 0.469544 |
| 5beta-Chol-11-en-24-oic Acid | 0.636026 | 0.238802 | 1.693996 | 0.365264 |
| N-Acetylneuraminic acid | 0.6563 | 0.233121 | 1.847665 | 0.425186 |
| Pradofloxacin | 0.864036 | 0.426692 | 1.749642 | 0.684767 |
| 6,8-Dihydroxypurine | 1.247755 | 0.727745 | 2.139338 | 0.421019 |
| MG(18:1(11Z)/0:0/0:0) | 0.837529 | 0.319682 | 2.194224 | 0.718245 |
| L-Aspartate-semialdehyde | 1.388898 | 0.717262 | 2.689445 | 0.329887 |
| Silyhermin | 0.380286 | 0.097855 | 1.477875 | 0.16272 |
| diginatin | 0.845583 | 0.312079 | 2.291116 | 0.741544 |
| biliverdin-IX-alpha | 0.860719 | 0.444816 | 1.665494 | 0.656078 |
| 4-Hydroxy-5-(dihydroxyphenyl)-valeric acid-O-methyl-O-sulphate | 0.762134 | 0.365087 | 1.590988 | 0.469454 |
| Deoxyinosine | 0.234294 | 0.037729 | 1.454967 | 0.119351 |
| Glycerophosphoinositol | 0.637091 | 0.274324 | 1.479581 | 0.294317 |
| Umbelliferyl Arachidonate | 1.09686 | 0.627316 | 1.917854 | 0.745715 |
| O-Ureidohomoserine | 0.34549 | 0.042998 | 2.776012 | 0.31749 |
| 2,4-Thiazolidinedicarboxylic acid, 2-methyl- | 0.461147 | 0.163744 | 1.298718 | 0.142868 |
| (5R,6R)-3-(2-Acetamidoethylsulfanyl)-6-ethyl-7-oxo-1-azabicyclo[3.2.0]hept-2-ene-2-carboxylic acid | 1.542788 | 0.924551 | 2.574434 | 0.096978 |
| SERATRODAST | 1.036951 | 0.589206 | 1.824944 | 0.899881 |
| O-Succinyl-L-homoserine | 0.565481 | 0.157438 | 2.031073 | 0.382204 |
| **CE(6 keto-PGF1alpha)** | 1.451667 | 1.002032 | 2.103063 | 0.048759 |
| OXYQUINOLINE | 0.951477 | 0.489905 | 1.847926 | 0.883241 |
| 4-Amino-4-deoxyarabinose | 0.633069 | 0.176562 | 2.269886 | 0.482845 |
| (1S,2S,3S,4R)-3-(1-Acetamido-2-ethylbutyl)-4-(diaminomethylideneamino)-2-hydroxycyclopentane-1-carboxylic acid | 1.019166 | 0.570897 | 1.819416 | 0.948807 |
| Ascorbic acid | 0.699894 | 0.250556 | 1.955061 | 0.495988 |
| Phoxim | 0.90344 | 0.449328 | 1.8165 | 0.775682 |
| thioquinone | 0.461032 | 0.131635 | 1.614691 | 0.225996 |
| gamma-Glutamylglutamic acid | 1.128081 | 0.603381 | 2.109062 | 0.705801 |
| Coniferaldehyde | 0.493044 | 0.132566 | 1.833747 | 0.291342 |
| SPARSOMYCIN | 0.660432 | 0.237284 | 1.838175 | 0.426998 |
| PA(PGJ2/18:4(6Z,9Z,12Z,15Z)) | 1.610297 | 0.941553 | 2.754023 | 0.081858 |
| Palmitoleic acid | 0.351678 | 0.051907 | 2.382653 | 0.28437 |
| Flutamide | 0.907908 | 0.450964 | 1.827858 | 0.786697 |
| S-(2-Carboxyethyl)-L-cysteine | 0.459212 | 0.13482 | 1.564131 | 0.213285 |
| Alanylserine | 1.101003 | 0.511388 | 2.370428 | 0.805737 |
| 8-Hydroxymianserin | 1.381376 | 0.94639 | 2.016293 | 0.094052 |
| Tertatolol | 0.752458 | 0.345852 | 1.637099 | 0.473308 |
| (-)-Cuminone A | 0.919645 | 0.467063 | 1.810778 | 0.808529 |
| 1-O-Hexadecyl-sn-glycero-3-phosphocholine | 0.620472 | 0.212139 | 1.814786 | 0.383425 |
| isopropyl unoprostone | 1.334793 | 0.740214 | 2.40697 | 0.33707 |
| PS(O-16:0/0:0) | 1.060316 | 0.615237 | 1.827376 | 0.832976 |
| Glyceryl monolinoleate | 0.95149 | 0.43196 | 2.095874 | 0.901777 |
| N-Stearoyl Cysteine | 0.537802 | 0.158244 | 1.827756 | 0.320349 |
| Cysteine-glutathione disulfide | 0.677274 | 0.263691 | 1.739537 | 0.418132 |
| 3'-Deaminofusarochromanone | 1.142126 | 0.648562 | 2.011298 | 0.645322 |
| Uridine diphosphate glucuronic acid | 0.6592 | 0.267046 | 1.627227 | 0.366047 |
| Hippuryl-glycyl-glycine | 1.132051 | 0.652501 | 1.96404 | 0.65906 |
| N1,N12-Diacetylspermine | 0.777595 | 0.369197 | 1.637754 | 0.508039 |
| 3'-Deoxythymidine | 1.560089 | 0.790132 | 3.080342 | 0.200081 |
| Hexazinone | 0.587977 | 0.240288 | 1.438761 | 0.244756 |
| Foetidin | 1.570986 | 0.870374 | 2.835561 | 0.133826 |
| Valylserine | 0.899265 | 0.425766 | 1.899345 | 0.780759 |
| gamma-Glutamylcysteine | 0.506464 | 0.150936 | 1.699436 | 0.270718 |
| 5'-Methylthioadenosine | 0.532404 | 0.132398 | 2.140928 | 0.374643 |
| Glutaminylproline | 1.407188 | 0.867427 | 2.282817 | 0.166417 |
| MG(24:6(6Z,9Z,12Z,15Z,18Z,21Z)/0:0/0:0) | 1.128327 | 0.641545 | 1.984462 | 0.67513 |
| Phenylalanylasparagine | 1.362831 | 0.642502 | 2.890741 | 0.419734 |
| 9-Octadecenoic acid (9Z)-, (2-hydroxy-2-oxido-1,2-oxaphospholan-4-yl)methyl ester | 1.31953 | 0.758244 | 2.296305 | 0.326637 |
| Glycyl-Isoleucine | 1.300192 | 0.631725 | 2.676004 | 0.475965 |
| 3,6-Dioxo-5alpha-cholan-24-oic Acid | 1.271422 | 0.763976 | 2.11592 | 0.355472 |
| Pisumionoside | 0.781998 | 0.365508 | 1.67307 | 0.526284 |
| Cholesterol sulfate | 0.439949 | 0.123758 | 1.563982 | 0.204495 |
| 6-Feruloylglucose 2,3,4-trihydroxy-3-methylbutylglycoside | 0.647248 | 0.213166 | 1.965272 | 0.442674 |
| Serdexmethylphenidate | 0.986218 | 0.519102 | 1.873672 | 0.966194 |
| beta-heptenoic acid | 0.502928 | 0.142575 | 1.774065 | 0.285235 |
| PA(O-20:4(5Z,8Z,11Z,14Z)/2:0) | 1.145549 | 0.65 | 2.018896 | 0.638362 |
| DGTS(16:0/20:4(5Z,8Z,11Z,14Z) | 0.526952 | 0.149415 | 1.858439 | 0.319134 |
| TMS | 0.693634 | 0.293631 | 1.638549 | 0.404247 |
| Phenylalanylaspartic acid | 1.110431 | 0.525786 | 2.345168 | 0.783615 |
| Cytidine-5'-diphosphocholine | 0.208099 | 0.036328 | 1.192071 | 0.077955 |
| Uridine diphosphate-N-acetylglucosamine | 0.308035 | 0.078299 | 1.211843 | 0.091986 |
| 6,10,14-Trimethyl-5,9,13-pentadecatrien-2-one | 1.024999 | 0.547908 | 1.917519 | 0.938411 |
| 1-(11Z-eicosenoyl)-glycero-3-phosphate | 1.094942 | 0.607425 | 1.973739 | 0.762879 |
| Aspartyl-Methionine | 0.825032 | 0.36873 | 1.846007 | 0.639732 |
| Benzoylcarnitine | 1.459421 | 0.843146 | 2.526147 | 0.176864 |
| Sar-Arg-Val-Tyr-Val-His-Pro-Ala | 1.035532 | 0.587164 | 1.826281 | 0.903995 |
| 7b-Hydroxy-3-oxo-5b-cholanoic acid | 0.458379 | 0.135043 | 1.555887 | 0.210924 |
| Adrenoyl ethanolamide | 0.568757 | 0.209624 | 1.543169 | 0.267831 |
| alpha-Linolenic acid | 0.724201 | 0.260608 | 2.012475 | 0.536042 |
| N2-Acetylornithine | 0.787861 | 0.30821 | 2.01397 | 0.618539 |
| PIP(PGJ2/16:0) | 1.005638 | 0.514036 | 1.967385 | 0.9869 |
| Methyl l-phenylalaninate | 0.440442 | 0.096792 | 2.004176 | 0.288844 |
| (S)-2,3-Dihydro-3,5-dihydroxy-2-oxo-3-indoleacetic acid 5-glucoside | 0.666165 | 0.255355 | 1.737878 | 0.406362 |
| Betaxolol | 0.492695 | 0.102387 | 2.370886 | 0.377208 |
| 2-O-ethyl PAF C-16 | 0.964151 | 0.515934 | 1.801754 | 0.908892 |
| (2R,6S)-2,6-Diaminoheptanedioic acid | 0.76376 | 0.194992 | 2.991551 | 0.698841 |
| 2-succinyl-5-enolpyruvyl-6-hydroxy-3-cyclohexene-1-carboxylate | 0.409965 | 0.126183 | 1.331966 | 0.138032 |
| 5-Hydroxy-3-[[5-(4-nitrophenyl)furan-2-yl]methylideneamino]-1H-imidazol-2-one | 0.437672 | 0.137199 | 1.396203 | 0.162696 |
| L-Theanine | 1.436967 | 0.690771 | 2.989232 | 0.332014 |
| Prolyl-Valine | 1.35045 | 0.664711 | 2.743619 | 0.406133 |
| Ergothioneine | 0.517263 | 0.140451 | 1.905009 | 0.321664 |
| Potassium phosphate (K(H2PO4)) | 0.306931 | 0.06452 | 1.460123 | 0.137732 |
| Glycyltyrosine | 1.320807 | 0.6743 | 2.587172 | 0.417287 |
| 2-[[3-Cyclohexyl-1-[2-[3-(diaminomethylideneamino)propylcarbamoyl]piperidin-1-yl]-1-oxopropan-2-yl]amino]acetic acid | 0.924373 | 0.484197 | 1.764706 | 0.8116 |
| DG(22:5n6/0:0/22:5n6) | 1.208764 | 0.654374 | 2.232839 | 0.544819 |
| PC(17:1(9Z)/0:0) | 0.818177 | 0.340789 | 1.964302 | 0.653367 |
| PS(22:6(4Z,7Z,10Z,13Z,16Z,19Z)/0:0) | 0.27591 | 0.040976 | 1.857832 | 0.185709 |
| Phenylalanyl-Glycine | 1.135166 | 0.499229 | 2.581183 | 0.762282 |
| H-beta-Ala-Tyr-OH | 1.308216 | 0.63792 | 2.682826 | 0.463451 |
| (E,E)-3,7,11-Trimethyl-2,6,10-dodecatrienyl octanoate | 0.845338 | 0.42022 | 1.70053 | 0.637538 |
| PS(22:4(7Z,10Z,13Z,16Z)/0:0) | 0.799228 | 0.272546 | 2.343696 | 0.683066 |
| PC(0:0/14:0) | 0.983285 | 0.308313 | 3.135939 | 0.977275 |
| Sterol | 1.322061 | 0.732093 | 2.387463 | 0.354531 |
| LysoPE(0:0/20:1(11Z)) | 1.381521 | 0.790667 | 2.413911 | 0.256354 |
| DG(20:5(5Z,8Z,11Z,14Z,17Z)/22:6(4Z,7Z,10Z,13Z,16Z,19Z)/0:0) | 0.212837 | 0.009434 | 4.80174 | 0.330483 |
| 1-(9Z-Eicosenoyl)-sn-glycero-3-phosphocholine | 0.935604 | 0.488818 | 1.790757 | 0.840733 |
| Phenylalanylthreonine | 1.411591 | 0.690624 | 2.885201 | 0.344606 |
| Valylvaline | 0.837532 | 0.336651 | 2.083641 | 0.703003 |
| S-Adenosylmethionine | 0.623052 | 0.106823 | 3.634002 | 0.598995 |
| geldanamycin | 0.866192 | 0.368775 | 2.034541 | 0.741619 |
| Bryaquinone | 1.198285 | 0.658895 | 2.179234 | 0.553318 |
| PI(20:3(8Z,11Z,14Z)/0:0) | 0.381031 | 0.085843 | 1.691275 | 0.204476 |
| Ophthalmic acid | 1.140205 | 0.5553 | 2.341201 | 0.720761 |
| Pantoprazole sulfide | 0.620092 | 0.083567 | 4.60126 | 0.64026 |
| Phenylalanyl-Gamma-glutamate | 1.39533 | 0.682914 | 2.850941 | 0.360823 |
| Seryltyrosine | 1.0793 | 0.527144 | 2.209811 | 0.834665 |
| Deoxycholyl-L-dopa | 0.911347 | 0.460106 | 1.805134 | 0.790077 |
| 19-Methylhenicosanoylcarnitine | 0.948427 | 0.510225 | 1.762974 | 0.867054 |
| Oxidized glutathione | 1.097761 | 0.487325 | 2.472844 | 0.821893 |
| 1-Propenyl 1-(1-propenylsulfinyl)propyl disulfide | 0.510551 | 0.143757 | 1.813218 | 0.298504 |
| Glycyl-Phenylalanine | 1.349874 | 0.689778 | 2.641659 | 0.381137 |
| LysoPC(16:1(9Z)/0:0) | 0.388527 | 0.0631 | 2.392272 | 0.308003 |
| PC(P-19:1(12Z)/0:0) | 1.271185 | 0.80453 | 2.008517 | 0.303911 |
| **Diflucortolone valerate** | 0.042078 | 0.002156 | 0.821427 | 0.036644 |
| Kolaflavanone | 1.217003 | 0.659668 | 2.245213 | 0.529655 |
| (+)-Lysergic acid | 1.160259 | 0.492851 | 2.731456 | 0.733655 |
| Guanidoacetic acid | 0.740404 | 0.276637 | 1.981655 | 0.549597 |
| Isoleucyl-Gamma-glutamate | 1.206063 | 0.652892 | 2.227916 | 0.549594 |
| hydroxyisovaleroyl carnitine | 0.375716 | 0.063653 | 2.217669 | 0.27983 |
| Chelerythrine | 0.082008 | 0.003157 | 2.130536 | 0.132364 |
| Manbeta1-4Glcbeta-Cer(d18:1/16:0) | 0.756678 | 0.296912 | 1.928391 | 0.559121 |
| Lacto-N-biose I | 0.565612 | 0.105035 | 3.045822 | 0.507087 |
| ALANYL-dl-PHENYLALANINE | 1.406132 | 0.77592 | 2.548213 | 0.26118 |
| Leucylphenylalanine | 0.847143 | 0.302995 | 2.368525 | 0.75183 |
| N-(3-Amino-3-oxopropyl)-L-valine | 0.971248 | 0.438002 | 2.153693 | 0.942761 |
| Serylphenylalanine | 1.064174 | 0.516528 | 2.192458 | 0.866069 |
| L-Cystine | 0.033491 | 0.000212 | 5.299585 | 0.188664 |
| Teneligliptin | 1.539303 | 0.79366 | 2.985477 | 0.201886 |
| Lenticin | 0.865133 | 0.411993 | 1.816669 | 0.701915 |
| Leucyl-leucine | 0.900923 | 0.372241 | 2.180475 | 0.817035 |
| 1-(2-methoxy-6Z-heptadecenyl)-sn-glycero-3-phosphoserine | 0.364427 | 0.022124 | 6.002746 | 0.480081 |
| PS(20:4(5Z,8Z,11Z,14Z)/0:0) | 0.460065 | 0.060962 | 3.472024 | 0.451514 |
| Cysteinyl-Serine | 1.301167 | 0.851032 | 1.989391 | 0.224246 |
| PE(17:1(9Z)/0:0) | 0.883158 | 0.405199 | 1.924902 | 0.754611 |
| Leucyl-Glutamate | 0.916313 | 0.375243 | 2.237562 | 0.847847 |
| Biotinyl-5'-AMP | 0.903345 | 0.480517 | 1.698237 | 0.752291 |
| **N6-Succinyl Adenosine** | 0.028866 | 0.000974 | 0.85534 | 0.040332 |
| Stearoylethanolamide | 1.160533 | 0.608305 | 2.214079 | 0.651464 |
| 2-O-beta-D-Glucopyranuronosyl-D-mannose | 0.38704 | 0.031582 | 4.7432 | 0.457835 |
| Sulfamide, N,N-dimethyl-N'-((8alpha)-6-propylergolin-8-yl)- | 1.205051 | 0.697675 | 2.08141 | 0.503552 |
| Histidylisoleucine | 1.616477 | 0.836706 | 3.122959 | 0.152904 |
| **N1,N8-Diacetylspermidine** | 2.140986 | 1.068327 | 4.290653 | 0.031848 |
| Glutamylleucine | 0.941838 | 0.403161 | 2.200261 | 0.889913 |
| Glutathione | 0.835183 | 0.313177 | 2.227274 | 0.718939 |
| Adenylsuccinic acid | 0.226619 | 0.011757 | 4.368264 | 0.325443 |
| LysoPE(0:0/16:1(9Z)) | 0.22468 | 0.009659 | 5.226567 | 0.3524 |
| Glutathionate(1-) | 0.853447 | 0.333018 | 2.187188 | 0.741368 |
| 13-(3-Pentyloxiran-2-yl)trideca-2,4,6-trienoic acid | 0.728487 | 0.28085 | 1.889597 | 0.514784 |
| HR, hazard ratio; CI, confidence interval |  |  |  |  |

**Table S3. Univariate Cox regression analysis for the association between altered gastric microbiota and OS.**

| **Variable** | **HR** | **Lower_CI** | **Upper_CI** | **P_Value** |
| --- | --- | --- | --- | --- |
| bacteriap25 | 0.783101 | 0.358763 | 1.709338 | 0.539292 |
| Chryseobacterium | 1.495748 | 0.965148 | 2.318052 | 0.071662 |
| Dubosiella | 1.2005 | 0.777542 | 1.853533 | 0.409611 |
| SM2D12 | 0.016483 | 0 | NOT REACH | 0.998282 |
| Nordella | 3.79E-11 | 0 | NOT REACH | 0.9974 |
| Subgroup_10 | 0.78732 | 0.434722 | 1.425907 | 0.430056 |
| Pseudomonas | 0.554217 | 0.221372 | 1.387513 | 0.207492 |
| Chryseolinea | 0.814528 | 0.414121 | 1.602081 | 0.552247 |
| Janibacter | 1.175623 | 0.865054 | 1.59769 | 0.301248 |
| Helicobacter | 1.156702 | 0.762197 | 1.755399 | 0.493967 |
| Sphingomonas | 1.089262 | 0.66052 | 1.796301 | 0.737623 |
| Acinetobacter | 0.460671 | 0.153021 | 1.38685 | 0.168088 |
| Ralstonia | 0.692517 | 0.283038 | 1.694398 | 0.420909 |
| Sediminibacterium | 0.921006 | 0.524077 | 1.618566 | 0.774841 |
| Cryobacterium | 0.014807 | 0 | NOT REACH | 0.998351 |
| Granulicatella | 0.99414 | 0.537754 | 1.837857 | 0.985044 |
| Bergeyella | 1.001701 | 0.468988 | 2.139512 | 0.996498 |
| Colidextribacter | 0.944397 | 0.556785 | 1.60185 | 0.83194 |
| Bradyrhizobium | 1.185286 | 0.765493 | 1.835292 | 0.446058 |
| Harryflintia | 0.801836 | 0.367158 | 1.751128 | 0.57947 |
| Porphyrobacter | 0.103491 | 0 | NOT REACH | 0.998014 |
| Lachnospiraceae_UCG-004 | 1.79E-30 | 0 | NOT REACH | 0.99658 |
| **[Eubacterium]_ventriosum_group** | 1.370561 | 1.00958 | 1.860613 | 0.04327 |
| Lachnoanaerobaculum | 0.804829 | 0.363227 | 1.783318 | 0.592726 |
| Fusobacterium | 0.474131 | 0.069256 | 3.245933 | 0.447045 |
| Gemella | 0.123501 | 0.000589 | 25.8916 | 0.443156 |
| UCG-010 | 1.211048 | 0.836978 | 1.752299 | 0.309693 |
| Anaerotruncus | 0.335273 | 0.053252 | 2.11086 | 0.244376 |
| F082 | 1.187795 | 0.886641 | 1.591238 | 0.248693 |
| Parasutterella | 1.180265 | 0.721577 | 1.93053 | 0.509141 |
| Prevotella | 1.357047 | 0.713462 | 2.581186 | 0.351995 |
| Herbaspirillum | 1.035414 | 0.669166 | 1.602117 | 0.875831 |
| Acidovorax | 0.840221 | 0.450255 | 1.567937 | 0.584418 |
| IMCC26256 | 0.985515 | 0.609705 | 1.592969 | 0.952511 |
| Fenollaria | 1.173911 | 0.856001 | 1.609891 | 0.319711 |
| Carboxylicivirga | 0.089502 | 0 | NOT REACH | 0.998231 |
| **AKAU4049** | 1.711984 | 1.063229 | 2.756593 | 0.026951 |
| [Eubacterium]_oxidoreducens_group | 1.006659 | 0.599449 | 1.690492 | 0.979979 |
| Streptococcus | 0.886704 | 0.432353 | 1.818521 | 0.742825 |
| Intestinimonas | 1.160345 | 0.753478 | 1.786915 | 0.499626 |
| Succinivibrio | 0.753221 | 0.341948 | 1.659144 | 0.481827 |
| HR, hazard ratio; CI, confidence interval | | | |  |
